# Classifying short genomic fragments from novel lineages using composition and homology

**DOI:** 10.1186/1471-2105-12-328

**Published:** 2011-08-09

**Authors:** Donovan H Parks, Norman J MacDonald, Robert G Beiko

**Affiliations:** 1 Faculty of Computer Science, Dalhousie University, 6050 University Avenue, Halifax, Nova Scotia, Canada

## Abstract

**Background:**

The assignment of taxonomic attributions to DNA fragments recovered directly from the environment is a vital step in metagenomic data analysis. Assignments can be made using *rank-specific *classifiers, which assign reads to taxonomic labels from a predetermined level such as named species or strain, or *rank-flexible *classifiers, which choose an appropriate taxonomic rank for each sequence in a data set. The choice of rank typically depends on the optimal model for a given sequence and on the breadth of taxonomic groups seen in a set of close-to-optimal models. Homology-based (*e.g*., LCA) and composition-based (*e.g*., PhyloPythia, TACOA) rank-flexible classifiers have been proposed, but there is at present no hybrid approach that utilizes both homology and composition.

**Results:**

We first develop a hybrid, rank-specific classifier based on BLAST and Naïve Bayes (NB) that has comparable accuracy and a faster running time than the current best approach, PhymmBL. By substituting LCA for BLAST or allowing the inclusion of suboptimal NB models, we obtain a rank-flexible classifier. This hybrid classifier outperforms established rank-flexible approaches on simulated metagenomic fragments of length 200 bp to 1000 bp and is able to assign taxonomic attributions to a subset of sequences with few misclassifications. We then demonstrate the performance of different classifiers on an enhanced biological phosphorous removal metagenome, illustrating the advantages of rank-flexible classifiers when representative genomes are absent from the set of reference genomes. Application to a glacier ice metagenome demonstrates that similar taxonomic profiles are obtained across a set of classifiers which are increasingly conservative in their classification.

**Conclusions:**

Our NB-based classification scheme is faster than the current best composition-based algorithm, Phymm, while providing equally accurate predictions. The rank-flexible variant of NB, which we term ε-NB, is complementary to LCA and can be combined with it to yield conservative prediction sets of very high confidence. The simple parameterization of LCA and ε-NB allows for tuning of the balance between more predictions and increased precision, allowing the user to account for the sensitivity of downstream analyses to misclassified or unclassified sequences.

## Background

Sequencing genetic material directly from environmental samples allows microbial ecology to be studied at the molecular level. Metagenomic techniques have been applied to microbial assemblages that live in natural environments [1-3], in association with humans or other animals [4,5], and in engineered habitats that support important industrial processes [6]. Although such sampling approaches cannot give a complete profile of genetic and biochemical diversity except for the simplest of communities, these techniques have nonetheless led to the discovery of proteins with novel functions, such as proteorhodopsin [7], and the exploration of functional diversity in different communities [8].

A critical problem in metagenome analysis is *taxonomic attribution*, the determination of which sequence reads or assemblies should be assigned to the genomes of specific microorganisms. Attribution is, however, a difficult problem when dealing with sequence reads generated by Sanger sequencing (500-1000 nucleotides) and much more so for reads generated by next-generation sequencing approaches (50-400 nucleotides). Further compounding the problem is the fact that most of the Earth's microbial biodiversity is unculturable at present [9] and much of the novelty in metagenomes corresponds to organisms that lack a representative sequenced genome from the same named species, genus, or higher rank [10]. To be effective, a taxonomic assignment algorithm must deal explicitly with unrepresented lineages, either by mapping such novelty to an appropriate taxonomic level (*e.g*., to the family level if no genus matches particularly well) or by using a criterion that allows some reads or assemblies to remain unassigned [11].

Supervised approaches to taxonomic assignment make use of reference genomes for classification purposes, and can be subdivided into *homology- *and *composition*-based methods, with a third class (*hybrid*) that combines these two. We also distinguish *rank-specific *approaches, which perform taxonomic assignments only to a particular rank (e.g., single genome, species, or genus), from *rank-flexible *approaches which can assign sequences to higher taxonomic or phylogenetic levels of classification if no clear preference is seen at more-precise levels.

Homology-based approaches such as best BLAST matching [12] capitalize on the conservation of long protein-coding sequences and shorter protein domain motifs to identify best sequence matches from a reference database. Such methods can yield precise results, but are fundamentally limited in that they cannot classify metagenomic sequences that lack detectable homology to the reference database and will be misled by false matches that arise from lateral gene transfer (LGT) or statistical artefacts. Taking the best BLAST match also forces a potentially overly precise assignment of the read to the best-matching genome. Rank-flexible classifiers have been developed to address this last limitation: the lowest common ancestor (LCA) algorithm implemented in MEGAN [11], the multiple taxonomic ranks (MTR) extension of LCA [13], the *q*-LCA method [14], Treephyler [15] and CARMA [16] all attempt to map sequences to an appropriate taxonomic level based on either the distribution of 'almost best' BLAST matches (*e.g*., LCA, MTR, *q*-LCA) or by building a reference phylogenetic tree and inserting the novel sequence to be assigned (*e.g*., Treephyler, CARMA). These latter approaches impose the additional requirement that homologous matches contain at least one conserved domain, which increases the accuracy of the approach at the cost of excluding many sequences from classification. BLAST matching also underpins the approach of PhymmBL [17] and the novel NB-BL approach we introduce below.

Composition-based approaches typically model a genome using the frequency profile of its constituent oligonucleotides (*n*-mers, with *n *typically between 2 and 10 nucleotides in length). A given metagenomic fragment will then be assigned to the genomes whose model best matches the fragment. The rank-specific Phymm classifier (Brady and Salzberg, 2009) uses interpolated Markov models to quantify the expected frequencies of variable-length *n*-mers up to *n *= 10. While many methods perform poorly on fragments in the sub-1000 bp range, Phymm achieves potentially useful accuracy levels on fragments of this length. The Naïve Bayes (NB) classifier has also been proposed for performing rank-specific classification of short metagenomic fragments [18-20] and is widely used for classifying 16S rRNA sequences [21]. However, the ability of NB to correctly classify fragments from novel lineages has not been adequately evaluated, as previous work only considered instances where exact strain or species matches are present within the reference database. Using an evaluation framework which simulates novel lineages at different taxonomic ranks, we demonstrate that NB generalizes well to unrepresented lineages and performs on par with Phymm. Interestingly, these methods substantially outperform a rank-specific nearest neighbour (NN) classifier.

While compositional variation at the genome level has been extensively documented [22-24], applying compositional approaches in a rank-flexible way often depends on the existence of compositional patterns that can discriminate at higher taxonomic ranks (*e.g*., class or phylum level). PhyloPythia [25] uses Support Vector Machines (SVMs) built at each taxonomic rank in a top-down fashion to determine the most appropriate rank at which to classify a fragment, while its successor, PhyloPythiaS [26], uses an ensemble of genome models to make rank-flexible predictions. Similarly to PhyloPythia, TACOA [27] uses an extension of the NN paradigm to classify fragments to the lowest taxonomic rank (LTR) with sufficient evidence to unambiguously assign the fragment to a single lineage. In this paper, we propose a simple rank-flexible extension of the NB classifier called ε-NB which outperforms PhyloPythiaS and TACOA.

Fundamental limitations of compositional convergence, incomplete sampling, and LGT prohibit the development of classifiers with perfect accuracy, but our goal is to develop a classifier that can make confident predictions at appropriate taxonomic levels, while removing uncertain fragments from classification. Table 1 presents the most prominent methods organized according to our scheme, and introduces three variants of a new hybrid, rank-flexible classifier that combine the best matches identified by an NB classifier with homology information derived from BLAST. Here we develop these new methods, and demonstrate their advantages in terms of classification performance and handling of uncertainty relative to other published approaches.

**Table 1:** Classification of supervised taxonomic assignment approaches

	Homology	Composition	Hybrid
Rank-specific	Best BLAST match [12]	**NN **(this paper) NB [18], Phymm [17]	**NB-BL **(this paper) PhymmBL [17]

Rank-flexible	LCA [11], MTR [13] *q*-LCA [14] Treephyler [15] CARMA [16]	**ε-NB **(this paper) PhyloPythia [25] PhyloPythiaS [26] TACOA [27]	**BLASTN + NB **(this paper) **BLASTN + ε-NB **(this paper) **LCA + NB **(this paper) **LCA + ε-NB **(this paper)

## Methods

### Test sets

#### Simulated test sets

Simulated test sets were derived from the complete bacterial and archaeal genomes available in the National Center for Biotechnology Information (NCBI) RefSeq database as of May 2010 [28]. Taxonomic information for each genome was obtained from the NCBI taxonomy database [29]. To evaluate the performance of different classifiers, we built a set comprising only genomes from genera represented by at least 3 distinctly named species. This filtered data set consists of 534 genomes from 334 species, 61 genera, 53 families, 34 orders, 20 classes, and 13 phyla with representatives from both the bacterial and archaeal domains. Simulated test sets were constructed by randomly sampling 100 fragments from each species with the probability of drawing a fragment from a contig (*i.e*., chromosome or plasmid) set proportional to its length. Three independent test sets with query fragments 200 bp, 400 bp, and 1000 bp in length were constructed.

#### Enhanced Biological Phosphorus Removal (EBPR)

Reads from the Madison, Wisconsin EBPR community sampled by Garcia Martín *et al*. were obtained from the NCBI Trace Archive [6]. This metagenome sample consists of 127,953 reads with an average length of 945 ± 142 bp. Reads were classified using all 1073 bacterial and archaeal genomes in the NCBI RefSeq database as of May 2010.

#### Glacier ice metagenome

The 1,076,539 pyrosequencing reads which comprise the glacier ice metagenome sampled by Simon *et al*. were obtained from the NCBI Sequence Read Archive [2]. These reads have an average length of 222 ± 59 bp. Reads were classified using all 1073 bacterial and archaeal genomes in the NCBI RefSeq database as of May 2010. In additional to pyrosequencing reads, 338 16S rRNA sequences were amplified by Simon *et al*. [2]. We classified these sequences using the RDP NB classifier (release 10, update 26) with a bootstrap cutoff of 60% [21].

### Leave-one-out evaluation

The ability of different classifiers to identify fragments from novel lineages was assessed using a leave-one-out testing procedure where genomes from entire lineages were removed from the training set. To ensure query fragments can be meaningfully classified to the taxonomic parent of a removed lineage, query fragments from lineages with no sister were removed from consideration. For example, when performing leave-one-out classification at the rank of genus all training genomes from the genus being evaluated were removed. However, if a given genus represented the only genus within its family, then query fragments from this genus were removed from the test set. After filtering query fragments that did not meet this criterion, the simulated test sets contained 7,500, 33,400, 7,500, 19,400, 25,000, 23,700, and 33,400 fragments when performing leave-one-out classification at the ranks of species to domain, respectively. The taxonomic groups considered when excluding lineages at different taxonomic ranks from the training set are given in Additional file 1, Table S1.

### Genomic nucleotide composition

Nucleotide composition was computed over the negative and positive strands of all contigs within a genome. All overlapping *n-*mers within a sequence *S *with length *L *were used to construct the feature vector ***w_S _***= [*w*_1_, *w*_2_, ..., *w_M_*], where *M *= *L*-*n*+1. Any *n-*mers containing characters other than A, C, G, or T were discarded. The feature vector for a genome, ***w***, was formed by combining the feature vectors from all contigs within the genome. Occurrence profiles were generated which indicate the number of times each of the *N *= 4^n ^*n-*mers were observed in ***w***. The frequency profile of a genome was obtained by dividing each element in its occurrence profile by the total number of *n-*mers in the genome.

### NB and ε-NB classifiers

An NB classifier calculates the posterior probability of a query fragment, *F*, originating from a genome, *G_i_*, using Bayes' rule:(1)

The NB classifier assumes each *n-*mer in a fragment is independent of all other oligonucleotides. Although this conditional independence assumption is violated in most applications, the resulting classifier typically performs well [30]. Given this assumption, the probability of a query fragment originating from genome *G_i _*is:(2)

The conditional probabilities of observing an *n-*mer, *w_j_*, given a genome, *G_i_*, are estimated from the occurrence profile of the genome. Our NB classifier differs slightly from previous formulations [18,19] as Laplace smoothing is used when estimating these conditional probabilities:(3)

where *f *(*w_j_|G_i_*) is the number of times *n-*mer *w_j _*is observed in genome *G_i _*and *M_i _*is the number of overlapping *n-*mers in genome *G_i_*. In this study, we consider a flat prior for *P*(*G_i _*) and assign each fragment to the genome with the maximum-likelihood estimate:(4)

A suitable *n*-mer length for use with our NB classifier was determined using a separate set of tuning reads extracted from the genomes within our simulated test set. Based on this evaluation (see Results), we decided to report results for *n *= 10 which represents a trade-off between performance and computational cost. This also facilitates a more direct comparison with Phymm which also uses *n-*mers with a maximum length of 10.

We also propose a rank-flexible ε-NB classifier where all genome models with a likelihood at most ε times smaller than the maximum-likelihood estimate influence the classification of a query fragment. A query fragment is classified as being from the LTR common to all genomes within this set and is considered *unclassified *at lower ranks. Except when explicitly studying the effect of varying ε values, all results are reported for ε = 10^5 ^which provides conservative classifications.

### NN and TACOA classifiers

Frequency profiles were calculated for all 534 training genomes in the data set and used as training vectors for the NN classifier when not within the lineage being evaluated. Given a query fragment to classify, its frequency profile was calculated and the Manhattan distance to each of the training vectors determined. A fragment is classified as being from the same strain as the closest training genome. All results were obtained using *n-*mers of length 4 which was found to be optimal based on preliminary experiments.

Diaz *et al*. proposed a kernelized NN algorithm for classifying genomic fragments known as TACOA [27]. In contrast to our NN classifier, TACOA allows all training vectors to contribute to the classification of a query fragment. Each training vector gives a weighted vote indicating the assignment of the query fragment with weights determined by a Gaussian kernel. This classifier is rank-flexible, as a query fragment is classified to the most specific taxonomic rank with sufficient votes to permit it to be unambiguously assigned into a single lineage.

Results for TACOA were obtained using the software provided by Diaz *et al*. [27]. We used the default parameters which were tuned by Diaz *et al*. for fragments of length 800 bp to 50 kbp. For short sequences (< 3 kbp), *n-*mers of length 4 were found to be optimal. Default parameters were used to generate all results.

### BLASTN and LCA classifiers

Evidence of homology between a query fragment and each reference genome was assessed using the NCBI implementation of BLASTN, version 2.2.23 [31]. The expectation value (E-value) was used to determine the most likely originating genome of a query fragment. When multiple genomes were found with the same top-scoring E-value, fragments were assigned to the LTR shared by these genomes. Fragments lacking a match with an E-value less than the specified threshold were considered unclassified at all taxonomic ranks.

Our ε-NB classifier is a compositional analogue to the LCA algorithm [11]. LCA assigns a query fragment to the LTR in common among all BLASTN hits with a bit score within a specified percentage, *p*, of the highest bit score. All results are reported with *p *= 15% as this is in the middle of the range suggested by Huson *et al*. [11]; the authors also suggest using bit scores to filter out hits to genomes with limited evidence of homology. We remove spurious hits using an E-value threshold since expectation values have a direct statistical interpretation. A maximum E-value threshold of 10^-5 ^is used for reporting hits with BLASTN. Although E-values for homologs will scale differently depending on nucleotide composition and the rate of evolution of a particular gene, hits with an E-value above this threshold are likely to correspond to false positives or true homologs from phylogenetically distant organisms that will not contribute to accurate classification of fragments.

### Hybrid, rank-flexible classifiers

A set of rank-flexible classifiers is obtained by classifying fragments to the LTR in common among the predictions from a composition and homology-based classifier. We propose four classifiers which provide increasingly stringent criteria for the assignment of fragments to taxonomically specific ranks: BLASTN+NB followed by BLASTN+ε-NB and LCA+NB, and finally LCA+ε-NB.

### Phymm, PhymmBL, and NB-BL classifiers

Phymm uses interpolated Markov models (IMMs) which identify variable-length, non-adjacent *n*-mer patterns within a fixed-length window that are characteristic of a given genome. The software provided by Brady and Salzberg [17] builds an IMM for each chromosome and plasmid within a genome. Since the other considered classifiers use genome-level models, we modified the Phymm software to build IMMs for full genomes. These genome-level models were found to result in slightly superior performance to those built for each contig (data not shown), likely as a result of our simulated test sets containing few query fragments from plasmids due to their short length. The default parameters consider overlapping windows of length 12 in order to build variable-length *n-*mers with a maximum length of 10. These parameters were used for all reported results.

PhymmBL scores were obtained by taking a linear combination of the log-likelihoods from IMMs and the logarithm of E-values from BLASTN using the empirically determined values specified in [17], Score = IMM - 1.2·ln(E) + 4.8. In accordance with the implementation of Brady and Salzberg, fragments without a BLASTN hit below an E-value of 10 are classified exclusively using the IMM log-likelihoods. Fragments with one or more hits to genomes with an E-value of 0.0 are assigned to the IMM with the largest log-likelihood among these genomes.

We evaluated a rank-specific NB-BL classifier analogous to PhymmBL. The empirically determined scaling constant used in the PhymmBL scoring function was multiplied by 10 to account for NB log-likelihoods being approximately 10 times larger than Phymm likelihoods (*i.e*., Score = NB - 12·ln(E) + 4.8).

### PhyloPythiaS

Patil *et al*. proposed using an ensemble of linear SVMs for assigning taxonomic attributions to genomic fragments [26]. This classifier is rank-flexible as fragments are assigned to the LTR where the majority of classifiers in the ensemble are still in agreement. Results for PhyloPythiaS were obtained using the software provided by Patil *et al*. [26]. Models for the 534 genomes in our simulated test set were built using the provided training scripts. By default, PhyloPythiaS considers an ensemble of three classifiers trained on sequences of equal or greater length than the query sequences. In order to provide PhyloPythiaS with accurate models for classifying fragments of length 1000, we considered models trained with the following sequence lengths (1000, 1100, 1200), (1000, 1250, 1500), (1000, 1500, 2000), and the default values of (1000, 2000, 3000). Results were similar for all four parameter settings. We report results for models trained on sequences of length (1000, 1100, 1200) as this maximizes the specificity of the classifier while still classifying nearly as many fragments as with the default values. Default settings were used for all other parameters.

### Measuring classification performance

The performance of each classifier was summarized using the sensitivity, specificity, false negative rate, and unclassified rate measures. These measures are commonly used to evaluate the performance of methods designed to classify metagenomic fragments [16,25-27] as they capture important relationships among the number of true positive (*TP*_i_), false positive (*FP*_i_), false negative (*FN*_i_) and unclassified (*U*_i_) assignments within each lineage *i*. The fragments from lineage *i *are denoted by *Z_i _*= *TP_i _*+ *FI_i _*+ *U_i. _*Sensitivity is the proportion of fragments from a lineage correctly assigned to it, *Sn_i _*= *TP_i_/Z_i_*; specificity is the proportion of fragments assigned to a lineage which are correct, *Sp_i _*= *TP_i_/*(*TP_i _+ FP_i_*); the false negative rate is the proportion of fragments from a lineage incorrectly assigned to a different lineage, *FNr_i _*= *FN_i_/Z_i_*; and the unclassified rate is the proportion of fragments from a lineage which could not be classified, *Ur_i _*= *U_i_/Z_i_*. Sensitivity is equivalent to the true positive rate and specificity is equal to 1 - false positive rate.

We report the *average *of these measures over all lineages at a given taxonomic rank (*e.g*., *Sn_avg _*= (1/*n*) Σ_i _*Sn_i_*) as opposed to the *absolute *value (*e.g*., *Sn_abs _*= Σ*_i _TP_i_*/Σ*_i _Z_i_*). Reporting the average performance is preferred as lineages are not evenly represented in our test set. For example, 231 of the 534 genomes are from the Proteobacteria phylum. A classifier could preferentially assign fragments to the Proteobacteria phylum and have relatively high absolute sensitivity and specificity, but low average sensitivity and specificity. As such, average measures give a more relevant indication of how well a classifier is performing. Nonetheless, a strong classifier should also have high absolute specificity and sensitivity so absolute measures are also provided where appropriate.

### Statistical results

STAMP v1.08 [32] was used to identify genera where the proportion of sequences assigned by NB and Phymm were significantly different. P-values were obtained using Fisher's exact test with the Šidák correction for multiple tests. Confidence intervals were calculated using the Newcombe-Wilson method. Pearson's *r *statistic and analysis of covariance results were obtained with R v2.7.2 [33].

## Results

### Impact of *n-*mer length on NB performance

**Figure 1:**
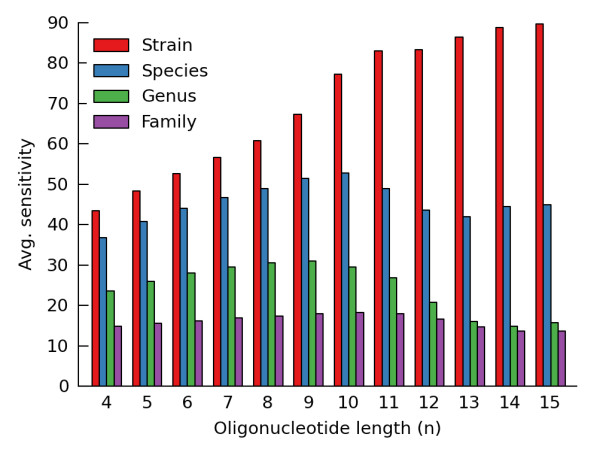
**Average sensitivity of NB classifiers with models built from oligonucleotides of varying length**. Performance was evaluated using a leave-one-out evaluation framework with lineages excluded from the training set at the strain, species, genus, or family rank. Fragments were considered correctly classified if they were assigned to the same order as their source genome. Results are for a simulated test set with 200 bp query fragments.

The performance of an NB classifier depends on the oligonucleotide length used to construct the compositional profile of a genome [18,19]. Using a leave-one-out evaluation framework, we assessed the performance of our NB classifier as query fragments become increasingly taxonomically distant from the training set (Figure 1). We confirm the results of Rosen *et al*. [19] that classification of unrepresented strains benefits from using oligonucleotides up to length 15. However, the optimal oligonucleotide length is 8 to 10 when query fragments originate from novel lineages at higher taxonomic ranks (Figure 1; Additional file 2, Tables S2-S5). As such, the recommendation to use oligonucleotides of length 15 is appropriate when it is known *a priori *that all query fragments are from species that are represented in the training genomes. When fragments may be from a novel genus or more diverse taxonomic rank, we recommend using oligonucleotides of length 10. However, the performance of our NB classifier is similar for *n *= 8 to 10.

### Performance of rank-specific classifiers

**Figure 2:**
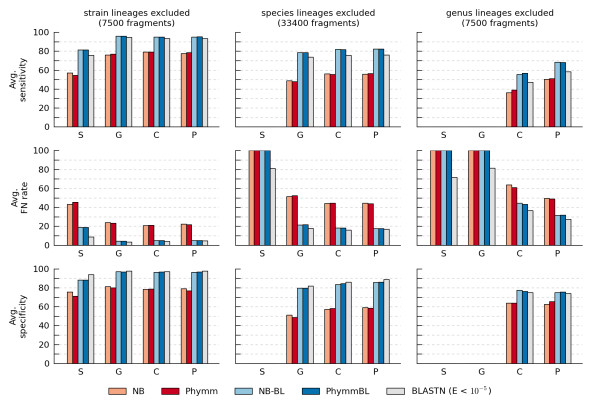
**Average classification performance of rank-specific classifiers on 200 bp query fragments**. Performance was evaluated using a leave-one-out framework with lineages excluded at the strain, species, or genus rank. Fragments were considered correctly classified if they were assigned to the species (S), genus (G), class (C), or phylum (P) of their source genome.

Six rank-specific classifiers (NN, NB, Phymm, NB-BL, PhymmBL, and BLASTN) were evaluated using a leave-one-out evaluation framework where lineages at different taxonomic ranks were excluded from the training set. Classification performance was evaluated on simulated test sets with query fragments of length 200 bp, 400 bp, and 1000 bp and lineages excluded at all ranks from strain to phylum. Although performance depends strongly on both fragment length and the rank of model exclusion, the relative performances of the classifiers were similar for the three different fragment lengths tested. In Figure 2, we report select results for 200 bp query fragments. We note that the performance measures do not always change monotonically when classifying fragments at more general taxonomic ranks as might be expected. This is a result of reporting *average *performance measures in Figure 2. Complete results, including absolute performance measures, are given in Additional file 3, Figures S1-S6.

The performance of Phymm and our NB classifier are similar despite the relaxed assumptions and flexibility of the Phymm models (Figure 2). For 200 bp fragments, the largest discrepancy in average sensitivity occurs when classifying fragments to a class with genus-level lineages excluded, where Phymm outperforms NB by 2.8% (39.1% vs. 36.3%). The second-largest discrepancy occurs when classifying reads to a species with strain-level lineages excluded, where NB outperforms Phymm by 2.5% (56.9% vs. 54.4%). Phymm and NB uniformly outperform the NN classifier (Additional file 3, Figures S1-S6). The homology-based BLASTN classifier performs substantially better than Phymm and NB: on query fragments of 200 bp with lineages excluded at the species level, its average sensitivity over all ranks is 20.2% and 19.7% higher than Phymm and NB, respectively.

The sensitivity of BLASTN can be increased by combining BLASTN E-values and Phymm likelihoods through the PhymmBL scoring function [17]. An analogous classifier can be constructed using the NB likelihoods which we coin NB-BL. The performance of PhymmBL and our proposed NB-BL classifier are nearly identical and both result in increased average sensitivity compared to BLASTN (Figure 2). However, this is at the expense of generally increasing the average false negative rate. For example, the average false negative rate of BLASTN when classifying fragments to a species with strain-level lineages excluded is 8.9% compared to 18.7% and 18.8% for PhymmBL and NB-BL, respectively. As a consequence of this increase in false negatives, the average specificity of PhymmBL and NB-BL is similar to that of BLASTN (Additional file 3, Figures S1-S3).

### Performance of rank-specific classifiers on specific taxonomic groups

We further investigated the taxonomic attributions made by NB, Phymm, and BLASTN on 200 bp fragments with species-level lineages excluded from the training set. Confusion matrices indicating the assignment of each fragment at the phylum and genus levels are given in Additional file 4 (Tables S6) and Additional file 5 (Table S8), respectively. The correlation between fragments *incorrectly *classified by these classifiers was measured using Pearson's *r *(*i.e*., the correlation of off-diagonal elements in the confusion matrix). At the phylum level all three classifiers are highly correlated: NB vs. Phymm = 0.99, NB vs. BLASTN = 0.93, Phymm vs. BLASTN = 0.93. Correlations were weaker at the genus level (NB vs. Phymm = 0.80, NB vs. BLASTN = 0.73, Phymm vs. BLASTN = 0.67). Genus-by-genus summaries of performance show large variations. *Desulfovibrio *exhibited the worst performance with sensitivity and specificity being less than 15% for both NB and Phymm, whereas other genera (e.g., *Pyrobaculum*) were classified with sensitivity and specificity both in excess of 75% (Additional file 4, Table S7). In spite of this wide variation, the accuracies of NB and Phymm track each other reasonably closely: the average difference in sensitivity and specificity across each pair of genera is 5.3% and 7%, respectively.

**Figure 3:**
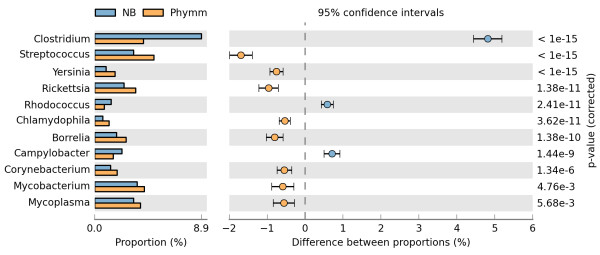
**Genera classified differently by NB and Phymm**. Genera where the proportion of fragments assigned by NB and Phymm differ by at least 0.5%. The left plot shows the proportion of assigned sequences while the right plot gives 95% confidence interval for the difference between proportions. Statistical significance was calculated with Fisher's exact test using the Šidák correction for multiple comparisons.

Although genus-level predictions were highly correlated between NB and Phymm, there are notable differences between these composition-based classifiers (Figure 3). In particular, the NB classifier preferentially assigns fragments to *Clostridium*: NB incorrectly assigned a total of 2241 fragments to members of genus *Clostridium *(including 883 to *C. botulinum *alone), compared to 840 for Phymm and 560 for BLAST (Additional file 5, Table S8). Conversely, *Streptococcus *is favoured by Phymm, with 924 fragments incorrectly assigned by this classifier to genus *Streptococcus*, as compared with only 464 by NB and 199 by BLAST. Apart from *Clostridium*, only *Rhodococcus *and *Campylobacter *are significantly (see Methods) overrepresented in NB predictions relative to Phymm (Figure 3). *Rhodococcus, Mycobacterium*, and *Corynebacterium *are members of suborder Corynebacterineae, and the latter two are overrepresented in Phymm predictions. The overrepresentation of *Clostridium *and *Campylobacter*, both of which consist of G+C-poor genomes, appears to result from fragments being preferentially assigned to other G+C-poor genera with relatively small genomes (< 1.5 Mb) such as *Mycoplasma, Rickettsia, Borrelia *and *Wolbachia *(Additional file 5, Table S8). The first three of these are again overrepresented among Phymm predictions. The preference of Phymm for genus *Streptococcus *is due largely to misclassifications of members of class Bacilli including *Listeria, Bacillus *and *Lactobacillus*, and low-G+C organisms such as *Clostridium *and *Mycoplasma*. Other genera that are overrepresented among Phymm predictions include *Yersinia*, which Phymm assigns as a label to many fragments from other Gammaproteobacteria, particularly *Shewanella*, and *Chlamydophila *from which no clear pattern of misclassifications emerges.

### Performance of rank-flexible classifiers

Rank-specific classifiers assign all query fragments to a specific genome within the training set. When classifying reads from a metagenomic sample, this represents a serious limitation as naturally occurring microbial communities typically contain numerous organisms with no sequenced representatives. The leave-one-out evaluation framework simulates this scenario, and demonstrates that no fragments can be classified correctly (false negative rate = 100%) if the classification rank is lower than the excluded rank (Figure 2). The BLASTN classifier is the lone exception, as it deems fragments with insufficient evidence of homology to any training set genome as unclassifiable and resolves multiple top-scoring hits in a rank-flexible manner.

**Figure 4:**
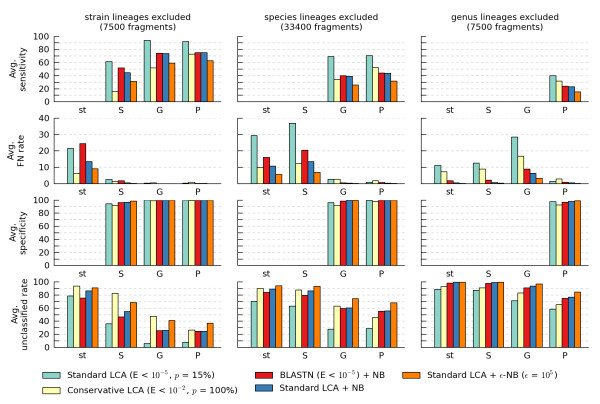
**Average classification performance of rank-flexible classifiers on 200 bp query fragments**. Performance was evaluated using a leave-one-out framework with lineages excluded at the strain, species, or genus rank. Fragments were considered correctly classified if they were assigned to the strain (st), species (S), genus (G), or phylum (P) of their source genome.

Rank-flexible classifiers address this limitation by attempting to assign a query fragment to the LTR in common between the fragment and its closest relative(s) within the training set. Five rank-flexible classifiers were evaluated on our simulated test sets with query fragments of length 200 bp, 400 bp, and 1000 bp using the leave-one-out framework with lineages excluded at all ranks from strain to phylum (Additional file 6, Figures S7-S12). Figure 4 gives results for 200 bp fragments with lineages excluded at the strain, species, and genus ranks as the general trends follow those observed under these conditions.

For typical parameters of E = 10^-5 ^and *p *= 15%, LCA results in a substantial reduction in the false positive rate at the expense of reduced sensitivity compared to BLASTN as a result of fragments being assigned to less taxonomically specific ranks. For example, when classifying fragments at the genus level with species-level lineages excluded from the training set, the average false negative rate decreases from 18.0% to 2.8% while the average sensitivity is reduced from 73.9% to 69.3% (Figures 2 and 4). Notably, at the species level the false negative rate decreases from 81.2% to 37.0% indicating a large portion of over-specific taxonomic assignments are removed by LCA.

A highly conservative homology-based rank-flexible classifier is obtained by assigning fragments to the LTR common to all hits with an E-value less than 10^-2^. We refer to this as *conservative *LCA, since it considers all BLAST matches with reasonable evidence of homology when assigning a taxonomic attribution to a fragment. Although this reduces the average false negative rate, a substantial number of fragments still remain assigned to an over-specific taxonomic rank (Figure 4). For classification at the species level with species-level lineages removed from the training set, the false negative rate remains over 10% as a result of over-specific classifications. To further reduce the number of over-specific classifications, the consensus between homology- and composition-based classifiers is considered.

Assigning fragments to the LTR in common between BLASTN and NB does not result in a substantial reduction in over-specific classifications under all conditions. In fact, the false negative rate at the strain level with strain-level lineages excluded from the training set is over 20% and higher than the false negative rate of the standard LCA classifier. This suggests that the LCA algorithm is relatively effective at identifying over-specific assignments when query fragments are from genomes where the closest relative in the training set is from the same species. In contrast, the BLASTN+NB classifier reduces the false negative rate compared to LCA when excluding lineages above the rank of strain.

Combining the standard LCA and NB classifiers results in a reduction in false positive classifications compared to standard LCA or BLASTN+NB. Notably, the average sensitivity of LCA+NB is nearly identical to BLASTN+NB. Compared to standard LCA, this hybrid classifier represents a trade-off among the sensitivity, false negative rate, specificity, and percentage of fragments classified. For applications requiring a set of fragments with high-confidence taxonomic attributions, the LCA algorithm can be combined with our proposed ε-NB classifier and fragments assigned to the LTR common to both classifiers. This allows a further reduction in the number of false positive classifications at the expense of sensitivity. This is a direct result of imposing stricter requirements on the evidence required to assign a fragment to more specific taxonomic ranks.

The benefit of a conservative rank-flexible classifier such as the proposed LCA+ε-NB classifier can be demonstrated by distinguishing between query fragments assigned to the *correct rank *and those assigned to the *correct lineage *only. Let *r *be the LTR in common between a query fragment and any genome in the training set. A fragment is classified to the correct rank if it is assigned to its lineage at rank *r*. It is classified to the correct lineage if it is assigned to its lineage at a more-specific rank than *r *(*i.e*., is an over-specific classification contained within *r*). An *incorrect *classification occurs when a fragment is assigned to a category at rank *r *or a rank more specific than *r *which is outside of its lineage. For rank-flexible classifiers, fragments may also be unclassified at rank *r*.

**Figure 5:**
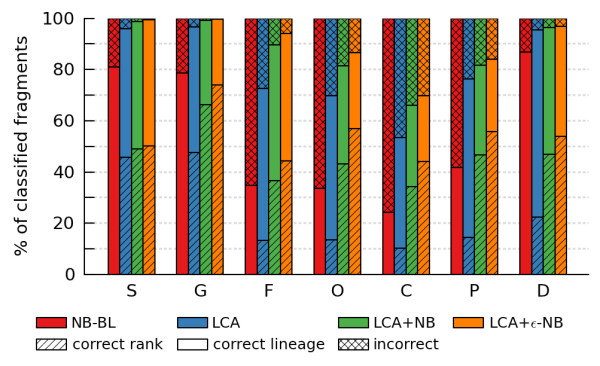
**Percentage of classified query fragments assigned to the correct rank, correct lineage, or incorrectly**. Each set of bars indicates the performance at a given rank when the child lineages of that rank are excluded from the training set. For example, results at the genus level are calculated with species level lineages excluded. Performance is reported at species (S), genus (G), family (F), order (O), class (C), phylum (P), and domain (D) ranks. The rank-specific NB-BL classifier always classifies query fragments at the strain level and as a result never assigns fragments to the correct rank. Results are reported for the 200 bp simulated test set. BLASTN and LCA results are for an E-value threshold of 10^-5^. The LCA classifiers use *p *= 15% and the ε-NB classifier uses ε = 10^5^.

Of the fragments classified at a given taxonomic rank, the percentage of fragments assigned to the correct rank increases and the percentage of fragments incorrectly classified decreases as one moves from the rank-specific NB-BL to the highly conservative rank-flexible LCA+ε-NB classifier. This holds true at all taxonomic ranks and for 200 bp, 400 bp, and 1000 bp fragments (Figure 5; Additional file 7, Figures S13-S14). Although this increased precision in taxonomic attribution is at the expense of an increased number of unclassified fragments at a given taxonomic rank (Figure 4), applications sensitive to over-specific or incorrect taxonomic attributions will benefit considerably from using one of the more-conservative rank-flexible classifiers.

### Comparisons to PhyloPythiaS and TACOA

PhyloPythiaS [26] and TACOA [27] are composition-based rank-flexible classifiers which make use of SVMs and the NN paradigm, respectively. Here we contrast their performance with the composition-based ε-NB, homology-based LCA, and hybrid LCA+NB rank-flexible classifiers (Table 2). We performed this comparison on 1000 bp query fragments as PhyloPythiaS and TACOA have been designed for contigs or longer fragments. All classifiers were trained on the 534-genome dataset which forms the basis of our leave-one-out evaluation framework.

**Table 2:** Performance of rank-flexible classifiers on 1000 bp query fragments

	Ge				Or				Cl				Ph			
	Sn	FNr	Sp	Ur	Sn	FNr	Sp	Ur	Sn	FNr	Sp	Ur	Sn	FNr	Sp	Ur
TACOA	8.3	0.7	91.6	91.0	15.0	1.3	86.1	83.6	18.5	1.2	93.7	80.3	31.2	4.3	87.9	64.4
PhyloPythiaS	35.2	1.6	94.3	63.2	48.8	3.5	92.7	47.7	56.9	4.1	92.8	39.0	58.7	5.6	93.5	35.7
**ε-NB (none)**^**a**^	**95.5**	**2.6**	**97.9**	**2.0**	**96.2**	**2.5**	**98.2**	**1.4**	**96.7**	**2.2**	**98.2**	**1.0**	**97.4**	**1.9**	**98.8**	**0.8**
**ε-NB (strain)**^**b**^	**92.6**	**4.5**	**97.5**	**3.0**	**92.2**	**5.1**	**96.7**	**2.8**	**92.3**	**5.0**	**96.4**	**2.7**	**92.1**	**5.2**	**97.5**	**2.8**
**ε-NB (species)**^**c**^	**71.0**	**17.0**	**84.5**	**12.0**	**73.0**	**16.6**	**86.2**	**10.4**	**75.4**	**15.7**	**87.6**	**8.9**	**74.8**	**16.8**	**89.7**	**8.4**
LCA (strain)^b^	96.2	0.8	99.3	3.0	95.8	0.9	99.1	3.3	95.4	0.8	99.3	3.9	95.2	0.9	99.3	4.0
LCA (species)^c^	82.9	4.1	95.5	13.0	85.2	2.4	97.5	12.4	85.6	1.8	98.0	12.6	85.9	1.7	98.7	1.7
**LCA+NB (strain)**^**b**^	**91.5**	**0.1**	**99.9**	**8.4**	**91.0**	**0.1**	**99.8**	**8.9**	**90.8**	**0.1**	**99.9**	**9.13**	**90.8**	**0.1**	**100**	**9.1**
**LCA+NB (species)**^**c**^	**66.9**	**0.5**	**99.2**	**32.6**	**69.1**	**0.3**	**99.5**	**30.6**	**70.9**	**0.2**	**99.8**	**28.9**	**70.4**	**0.4**	**99.9**	**29.2**

ε-NB results are for ε = 10^5^; LCA and LCA+NB results are for E-value < 10^-5^, p = 15%. Ge = genus, Or = order, Cl = class, Ph = phylum

^a ^no models excluded from training set; ^b ^strain-level lineages excluded from training set; ^c ^species-level lineages excluded from training set.

Both PhyloPythiaS and TACOA contain the source genome of all query fragments within their training set. Under these conditions, PhyloPythiaS was found to outperform TACOA. At the genus level, only 9% and 36.8% of fragments are classified by TACOA and PhyloPythiaS, respectively. In contrast, 98% of the fragments are classified by our ε-NB classifier while achieving higher average sensitivity and specificity. However, strong performance on the training set does not imply a high-quality classifier. To demonstrate that ε-NB, LCA, and LCA+NB generalize well, the performance of these classifiers with strain- and species-level lineages excluded from the training set was evaluated. Even with strain-level masking, the homology-based LCA classifier performs better than all the compositional classifiers. However, the proposed hybrid LCA+NB classifier permits a notable reduction in the average false negative rate at the expense of classifying fewer fragments. LCA+NB has the highest specificity among all considered classifiers.

### EBPR metagenome classification

EBPR communities are dominated by *Candidatus *Accumulibacter phosphatis, a novel lineage of Betaproteobacteria among the genomes in the NCBI RefSeq database. Reads from the Madison, Wisconsin EBPR community were classified using the BLASTN, NB-BL, LCA, and LCA+NB classifiers under two conditions: 1) when all genomes in NCBI's RefSeq database are used as the training set and 2) when *A. phosphatis *is removed from the training set. This allows the performance of these classifiers to be contrasted in the context of a novel lineage.

Of the 127,953 reads in the Wisconsin EBPR sample, *A. phosphatis *is the only BLASTN hit with an E-value below 10^-5 ^for 41,671 (32.6%) reads and the top BLASTN hit for 73,039 (57.1%) reads. Removal of *A. phosphatis *from the training set would ideally result in reads originating from this genome being classified as Betaproteobacteria and identified as 'unclassified' at more-specific taxonomic ranks. Within the rank-specific classification framework of PhymmBL or NB-BL, the 32.6% of reads having only a BLASTN hit to *A. phosphatis *are assigned to the top-scoring compositional model. This causes many fragments to be correctly assigned as Betaproteobacteria, but also results in a substantial increase in the proportion of reads classified as Alphaproteobacteria, Gammaproteobacteria, and Actinobacteria (Table 3). Since NB-BL classifies all reads, it fails to indicate that many of the EBPR reads are difficult to classify and thereby reveal that the community contains a novel lineage of Betaproteobacteria.

**Table 3:** Class-level assignment of EBPR reads with A. phosphatis included or excluded from the training set

	**BLASTN**^**a**^ (inc.)	**BLASTN**^**a**^ (excl.)	**NB-BL**^**a**^ (inc.)	**NB-BL**^**a**^ (excl.)	**LCA**^**b**^ (inc.)	**LCA**^**b**^ (excl.)	**LCA**^**b**^**+NB** (inc.)	**LCA**^**b**^**+NB** (excl.)
Betaproteobacteria	61.9	20.1	67.7	37.8	61.4	18.4	52.3	9.0
Gammaproteobacteria	5.4	8.2	10.0	19.8	5.1	7.2	1.9	2.5
Alphaproteobacteria	3.2	4.6	6.8	15.1	3.0	4.0	2.3	2.6
Deinococci	3.0	6.8	0.57	2.0	2.9	6.4	0.02	0.02
Actinobacteria	0.64	1.2	2.4	6.9	0.56	0.97	0.20	0.22

Other	1.1	1.6	12.5	18.4	0.84	1.6	73.2	0.26
Unclassified	24.8	57.5	0.0	0.0	26.2	61.4	42.3	85.4

^a ^E-value < 10^-5^; ^b ^E-value < 10^-5^, p = 15%

Results are shown for the 5 most abundant lineages identified by BLASTN.

We applied the rank-flexible LCA classifier to the EBPR sample with *p *set to 15%. This resulted in an average of 1.5 hits and 2.9 hits being considered by the LCA classifier with *A. phosphatis *included or excluded from the training set, respectively. This indicates that the top few hits for each fragment are substantially better than the remaining hits, and the similarity between the BLASTN and LCA results indicates high agreement at the class level among these top hits.

Our combined LCA+NB classifier results in a notable increase in the number of unclassified reads. Of the 42.3% of reads that are unclassified with *A. phosphatis *in the training set, 26.2% of these are the result of LCA having no hits with an E-value less than 10^-5 ^(24.8%) or having multiple hits that disagree on the classification of the fragment at the class level (1.4%). The remaining 16.1% are 'unclassified' due to disagreement between the NB and LCA classifications at the class level.

It is a significant challenge to correctly classify reads from genomes that are only distantly related to all training set genomes (*i.e*., at the class level in the case of *A. phosphatis*). Removal of *A. phosphatis *from the training set results in the LCA+NB classifier identifying an additional 43.1% of fragments as 'unclassified' at the class level. Although not ideal, this correctly indicates that the community contains a significant number of reads from an unrepresented lineage. Perhaps most importantly, with rank-flexible classifiers the removal of *A. phosphatis *from the training set does not substantially change the proportion of sequences assigned to classes other than Betaproteobacteria, in direct contrast to the rank-specific NB-BL classifier. Given the previous results, it is not surprising that the proportions reported by the LCA+NB classifier are least affected by removal of *A. phosphatis *from the training set.

### Glacier ice metagenome classification

**Figure 6:**
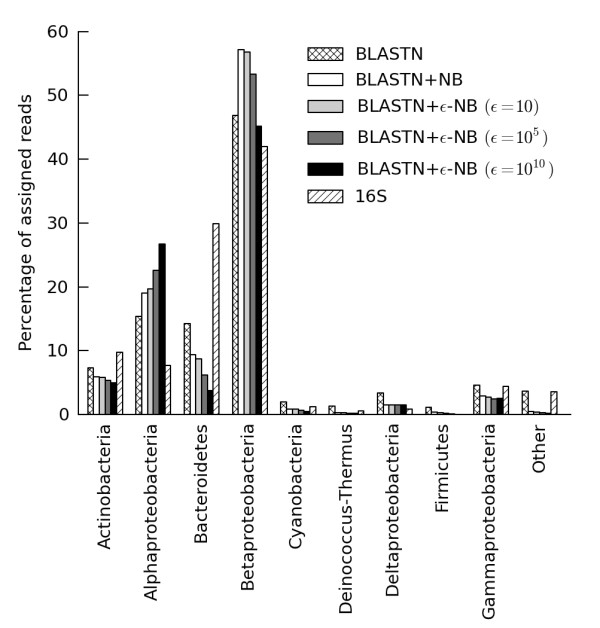
**Relative proportion of assigned reads from a glacier ice metagenome classified to different taxonomic groups**. Results are reported for a set of increasingly conservative classifiers along with a profile based on 338 amplified 16S rRNA sequences. Taxonomic groups represented by less than 1% of the assigned reads are collectively represented as 'Other'. BLASTN results are for an E-value threshold of 10^-5^.

Reads from the glacier ice metagenome sampled by Simon *et al*. [2] were classified using BLASTN, BLASTN+NB, and BLASTN+ε-NB in order to assess the stability of the percentage of assigned reads with increasingly conservative classifiers (Figure 6). The proportion of assigned reads was 43% for BLASTN, 29% for BLASTN+NB, 28% for BLASTN+ε-NB with ε = 10, 21% for BLASTN+ε-NB with ε = 10^5^, and 13% for BLASTN+ε-NB with ε = 10^10^. Setting the value of ε to 10, 10^5^, and 10^10 ^led to the inclusion of an average of 1.7, 12.1, and 42.1 models in the ε-interval, respectively. For comparison, CARMA provides taxonomic attributions for only 11% of the reads in this metagenome [2], while Treephyler classifies 15% of reads [15].

The obtained profiles on the metagenomic reads are in rough agreement with those obtained from the set of amplified 16S rRNA sequences, with Betaproteobacteria as the most abundant group, followed by Alphaproteobacteria, Bacteroidetes, and Actinobacteria in variable order, then Gammaproteobacteria and finally a set of low-abundance groups. Bacteroidetes assignments are more prevalent in the 16S profile (30%) than in the metagenomic profiles (4-14%) which are in agreement with results obtained using CARMA (8%) and Treephyler (9%) [15]. In contrast, assignments to Betaproteobacteria are less prevalent in the 16S profile (42%) than in the metagenomic profiles (45-57%) obtained with any of the classifiers considered here, but was found to be less abundant when classified with CARMA (24%) or Treephlyler (24%). Disagreements with the 16S profile may be the result of the relatively small number of 16S rRNA sequences which were amplified, variations in 16S copy numbers, variation in average genome sizes, or due to taxonomic biases in the reference databases used by the metagenomic classifiers. This latter issue may also explain the differences between the classifiers considered here (which use NCBI RefSeq bacterial and archaeal genomes as a reference database) and the results obtained with CARMA and Treephyler (which use PFAM as a reference database).

We also constructed taxonomic profiles using composition-based classifiers alone (Additional file 8, Figure S15). This is an attractive alternative given the computational efficiency of NB relative to BLAST. However, profiles obtained when using a composition-based classifier in isolation differ substantially from those obtained with a hybrid classifier: most notably, the highly represented Alphaproteobacteria and Betaproteobacteria decreased dramatically in frequency while many low-frequency groups increased. Changes to taxonomic profiles are expected as homology information is critical for accurate classification of short DNA reads (Figure 2).

### Influence of noise on compositional classifiers

**Figure 7:**
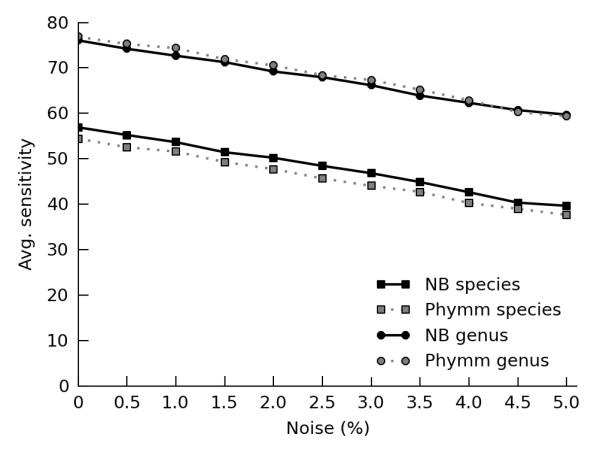
**Average sensitivity of NB and Phymm classifiers for varying levels of noise**. Results are reported for a simulated test set with 200 bp query fragments classified using a leave-one-out evaluation framework with lineages excluded at the strain level. Species and genus level classifications are shown.

Sequence noise can adversely affect the performance of a classifier. To evaluate the performance of compositional classifiers under noise, we artificially added noise to our simulated test set by randomly changing a certain percentage of bases (Figure 7). At 1% noise, 2 bases in a fragment of 200 bp will be randomly altered. The average sensitivity of NB and Phymm decreases gradually with increased noise. For classifications at the species level, the average sensitivity of NB is on average 2.3% higher than Phymm. The average decrease in sensitivity is 3.6% and 3.4% for every 1% increase in noise for NB and Phymm, respectively. For genus-level classifications, Phymm outperforms NB by an average of 0.7%, but decreases in performance by an average of 3.6% as opposed to 3.3% for every 1% increase in noise. An analysis of covariance indicates little evidence against the null hypothesis of equal slopes (p-value = 0.94 and 0.88 at the species and genus levels, respectively).

The application MetaSim [34] was used to investigate the influence of a more realistic noise model. MetaSim was used to generate 5,000 fragments with noise patterns characteristic of the Roche 454 sequencer (default parameters) from the 534 strains in our simulated test set. The median length of these fragments was 255 bp with 95% of the fragments between 235 bp and 312 bp. The results on these noisy fragments were compared to those obtained on the corresponding noise-free fragments. With strain-level lineages excluded from the training set, the average sensitivity at the species level for NB and Phymm decreased from 63.6% and 59.5% to 55.9% and 51.3%, respectively. For classifications at the genus level, average sensitivity for both NB and Phymm decreased from 81.4% to 74.9% and 74.7%, respectively. The NB and Phymm classifiers show similar sensitivity to noise under both the random and 454 noise models which supports the use of the computationally efficient NB classifier.

### Resource requirements of NB and Phymm

Our implementation of NB provides rapid model generation and classification of query fragments. Using a single core of an Apple Macbook with a 2.4 GHz Intel Core 2 Duo processor and 4 GB of RAM, NB models of all 1232 genomes in the NCBI RefSeq database as of December, 2010 can be built in less than 10 min in contrast to the 17 hours required to construct Phymm's IMMs. NB models also require a sixth of the storage space (5 GB vs. 30 GB). More critically, classification of one million 200 bp fragments using all 1232 genome models takes under 1.5 hours with NB and over 60 hours with Phymm.

## Discussion

Classifiers based on the NB paradigm perform as well as their Phymm counterparts, are conceptually simpler, and have reduced computational and memory requirements. The rank-specific PhymmBL and proposed NB-BL classifiers assign a query fragment to the genome with the highest support based on homology and compositional information, and provide a notable increase in the number of correctly classified fragments compared to BLAST [17]. This is accompanied by an increase in the number of incorrectly classified fragments and as a result the percentage of fragments correctly assigned to a lineage often decreases (Figure 2; Additional file 3, Figures S1-S6). Furthermore, these classifiers will invariably fail to inform us when a sample consists of one or more novel lineages as illustrated by our EBPR analysis. These limitations can be addressed by introducing an ε criterion which classifies a fragment to the LTR common to all models within the defined ε interval. When ε is small, few models are considered when classifying a fragment which tends to result in fragments being assigned to highly specific taxonomic ranks. In contrast, large ε values result in fragments being assigned to lower taxonomic ranks only when the best-scoring genome is a substantially better match to the fragment than all other genomes in the reference set.

LCA and the proposed ε-NB classifier are both ε-based classifiers. They substantially decrease the number of incorrect and over-specific classifications compared to their rank-specific counter-parts at the expense of some fragments remaining unclassified (Figures 2 and 4), and require the user to specify a choice of ε value. We have evaluated the proposed LCA+ε-NB classifier using an ε value of 10^5 ^in order to obtain a conservative set of taxonomic attributions. Although this may result in only a small subset of fragments being classified, most of these will be assigned to the correct rank (Figure 5). This high-specificity set of assignments is well-suited to applications that are sensitive to incorrect or over-specific classifications, such as the assignment of key metabolic pathways to community members or improving the accuracy of assemblies by pre-binning fragments into taxonomic groups.

Classification of a larger proportion of fragments, if desired, can be accomplished by reducing the stringency of taxonomic assignment (*i.e*., changing the E-value and *p *thresholds of LCA, or the ε threshold of ε-NB). Furthermore, by relaxing the requirement that *all *models within the set of plausible classifications agree, it may be possible to retain the benefits of ε-based classifier while correctly classifying additional fragments at each taxonomic rank. The rate of classification could also be increased by using the high-confidence set of classified fragments to initiate the recruitment of other fragments from the metagenome. For instance, the predicted enzymes in the high-confidence set could be used as seeds for metabolic pathway completion: if a fragment from taxonomic group X encodes enzyme A, then fragments encoding enzymes known to frequently co-occur with enzyme A are strong candidates for being assigned to taxonomic group X.

A recent extension to PhymmBL [35] fits a set of polynomial functions to the raw scores produced by PhymmBL in order to allow the user to specify a *linear *confidence score threshold. Although the expected proportion of correctly classified sequences for a given threshold value is dataset-specific and requires careful validation using a relevant dataset (*e.g*., a pseudo-metagenome), a doubling of this linear threshold is expected to double the number of, admittedly unknown, correct predictions. This strategy could be employed here in order to provide a threshold that is easier to interpret than directly setting ε. Evaluation of this proposed rank-flexible extension of PhymmBL is required before such an approach can readily be adopted. Nonetheless, it should be understood that rank-flexible thresholds such as the E-value for BLAST, the confidence score of PhymmBL, or the ε parameter for ε-NB are intentionally left as free parameters so they can be used in an exploratory sense to investigate the sensitivity of classifications, and set based on dataset specific auxiliary investigations which provide evidence for values suitable for individual research goals.

Depending on the application, it may also be desirable to restrict the set of available models. The genus-level misclassification results suggest strong biases in the misclassification of sequence fragments: for example, fragments from one genome with low G+C content tend to get assigned to other genomes with similarly extreme G+C. Perry and Beiko [24] observed that these genomes have a substantial number of regions with G+C content less than 20%, and such fragments were often misclassified due to their low complexity. However, only a subset of these genomes might be expected to occur in any particular habitat: for example, members of the *Mycoplasma, Rickettsia, Borrelia *and *Wolbachia *group identified above do not coexist, so the set of available models may be reasonably restricted based on a set of high-confidence assignments or a marker-gene study done in parallel. In other cases, a mismatch between taxonomy and phylogenetic relatedness may play a role, particularly with heterogeneous, polyphyletic genera such as *Clostridium*.

We conjecture that the strong performance of the rank-specific Phymm and NB classifiers relative to the NN classifier (Figure 2), and the rank-flexible ε-NB classifier relative to PhyloPythiaS and TACOA (Table 2) is a result of how absent *n-*mers are treated. Phymm, NB, and ε-NB determine the likelihood of a fragment originating for a reference genome by considering each of the *n-*mers observed in the fragment. The absence of a given *n-*mer in the query fragment does not contribute to this likelihood calculation. In contrast, the NN, PhyloPythiaS and TACOA classifiers compute the distance from a query fragment to each training genome which is a function of all possible *n-*mers. Likely as a consequence of this, these classifiers perform best for *n-*mers of length 4-6 which produce relatively dense feature vectors compared to Phymm and NB which consider *n-*mers of length 10. A dependence on dense feature vectors may also explain why PhyloPythiaS and TACOA are suited best for the classification of contigs or relatively long fragments (> 1 kbp).

## Conclusions

Phymm is the state-of-the-art for obtaining rank-specific classifications of short genomic fragments based on the compositional signal of source genomes. Despite the relative simplicity of NB, it performs as well as Phymm while providing a substantial reduction in running time. PhymmBL increases performance by making predictions based on a combination of Phymm model scores and BLASTN E-values. By combining BLASTN with the more computationally efficient NB classifier, we achieve accuracy scores that are indistinguishable from PhymmBL.

Rank-flexible classifiers address the more challenging problem of assigning an appropriate taxonomic rank to each query fragment that properly reflects the incomplete state of the training set and the confidence in a given prediction. Our ε-NB classifier is shown to outperform existing composition-based, rank-flexible classifiers (*e.g*., PhyloPythiaS, TACOA). Consistent with the pattern seen in rank-specific classifiers, the homology-based, rank-flexible LCA classifier outperforms NB. However, our proposed hybrid classifier which takes the intersection of the predictions produced by these two 'best-of-class' classifiers is shown to produce a high-confidence set of predictions in which classified fragments are rarely incorrect and often assigned to not just the correct lineage, but also the most appropriate taxonomic rank given the available set of reference genomes. The choice of an appropriate value for ε depends on the extent to which false-positive predictions can be tolerated: using our glacier ice metagenome, a user might choose a liberal value of ε (10^0 ^= best prediction only; or 10^1^) to generate a relatively large set of predictions with narrower taxonomic breadth, or a more conservative setting of 10^5 ^to 10^10 ^to make less-precise but higher-confidence assignments.

### Availability

Our cross-platform implementations of NB, NB-BL, ε-NB, LCA, and LCA+ε-NB are available under the GNU General Public License at http://kiwi.cs.dal.ca/Software/FCP. Instructions for constructing NB models are provided along with a script for building models of all genomes available through NCBI's RefSeq database.

## Competing interests

The authors declare that they have no competing interests.

## Authors' contributions

DHP developed and implemented the classifiers, provided experimental results, and wrote the initial draft of the manuscript. NJM collaborated in the development of the classifiers, implemented the leave-one-out evaluation framework, and provided auxiliary experimental results. RGB assisted with experimental design and the interpretation of results. All authors contributed to, read, and approved the final manuscript.

## Supplementary Material

Additional file 1 **Taxonomic groups used in leave-one-out evaluation**. List of taxonomic groups retained and removed when excluding lineages at different taxonomic ranks from the training set. Click here for file

Additional file 2 **Impact of *n-*mer length on NB performance**. Classification performance of NB classifiers with models built from oligonucleotides of varying length *n *and strain-level (Additional file 2, Table S2), species-level (Additional file 2, Table S3), genus-level (Additional file 2, Table S4), and family-level (Additional file 2, Table S5) lineages removed from the training set. The average sensitivity (*Sn*), false negative rate (*FNr*), and specificity (*Sp*) are reported for the 200 bp test set. Click here for file

Additional file 3 **Performance of rank-specific classifiers**. Average classification performance of rank-specific classifiers on 200 bp (Additional file 3, Figure S1), 400 bp (Additional file 3, Figure S2), and 1000 bp (Additional file 3, Figure S3) query fragments using a leave-one-out evaluation framework. Fragments were considered correctly classified if they were assigned to the species (S), genus (G), family (F), order (O), class (C), phylum (P), or domain (D) of their source genome. Corresponding results for absolute measures of performance are given in Additional file 3, Figures S4-S6. Click here for file

Additional file 4 **Classification results on specific taxonomic groups**. Classification results for NB, Phymm, and BLASTN on specific taxonomic groups detailed as a confusion matrix at the phylum level (Additional file 4, Table S6) and a summary table at the genus level (Additional file 4, Table S7). Results are reported for 200 bp query fragments with species-level lineages excluded from the training set. Click here for file

Additional file 5 **Genus-level confusion matrix**. Confusion matrix for NB, Phymm, and BLASTN at the genus level for 200 bp query fragments with species-level lineages excluded from the training set (Additional file 5, Table S8). Click here for file

Additional file 6 **Performance of rank-flexible classifiers**. Average classification performance of rank-flexible classifiers on 200 bp (Additional file 6, Figure S7), 400 bp (Additional file 6, Figure S8), and 1000 bp (Additional file 6, Figure S9) query fragments using a leave-one-out evaluation framework. Fragments were considered correctly classified if they were assigned to the species (S), genus (G), family (F), order (O), class (C), phylum (P), or domain (D) of their source genome. Corresponding results for absolute measures of performance are given in Additional file 6, Figures S10-S12. Click here for file

Additional file 7 **Percentage of classified query fragments assigned to the correct rank, correct lineage, or incorrectly**. Percentage of classified query fragments of length 400 bp (Additional file 7, Figure S13) and 1000 bp (Additional file 7, Figure S14) assigned to the correct rank, correct lineage, or incorrectly. Click here for file

Additional file 8 **Relative proportion of assigned reads from a glacier ice metagenome classified to different taxonomic groups**. Profiles obtained using composition-based classifiers are contrasted with profiles obtained with classifiers making use of homology information. Taxonomic groups represented by less than 1% of the assigned reads are collectively represented as 'Other'. The proportion of assigned reads was 43% for BLASTN, 85% for NB, 21% for BLASTN+ε-NB with ε = 10^5^, 46% for ε-NB with ε = 10^5^, 13% for BLASTN+ε-NB with ε = 10^10^, and 22% for ε-NB with ε = 10^10^. BLASTN results are for an E-value threshold of 10^-5^. Click here for file
