# Supplementary material for: Classifying short genomic fragments from novel lineages using composition and homology
Source: BMC Bioinformatics. 2011 Aug 9;12:328. doi: 10.1186/1471-2105-12-328 (PMC3173459; doi:10.1186/1471-2105-12-328)
Supplement: Additional file 1 — Taxonomic groups used in leave-one-out evaluation. List of taxonomic groups retained and removed when excluding lineages at different taxonomic ranks from the training set. [file 1471-2105-12-328-S1.PDF]

Additional File 1

Classifying short genomic fragments from novel lineages using composition and homology

Donovan H. Parks[1,§], Norman J. MacDonald[1,§], and Robert G. Beiko[1,*]

[1]Faculty of Computer Science, Dalhousie University, 6050 University Avenue, Halifax, Nova Scotia, Canada B3H 1W5

§ These authors contributed equally to this work.
[*] To whom correspondence should be addressed (beiko@cs.dal.ca).

Table S1. List of taxonomic groups retained and removed when excluding lineages at different taxonomic ranks from the training set. Included species are sorted and grouped at the genus level.

Lineage excluded	Retained taxonomic groups	Removed taxonomic groups
Species	334: **Acidovorax**:citrulli,ebreus,sp. JS42<br>**Anaeromyxobacter**:dehalogenans,sp. Fw109-5,sp. K<br>**Anaplasma**:centrale,marginale,phagocytophilum<br>**Arthrobacter**:aurescens,chlorophenolicus,sp. FB24<br>**Aster yellows witches-broom phytoplasma**<br>**Bacillus**:amyloliquefaciens,anthracis,cereus,clausii,cytotoxicus,halodurans,licheniformis, megaterium,pseudofirmus,pumilus,subtilis,thuringiensis,weihenstephanensis<br>**Bacteroides**:fragilis,thetaiotaomicron,vulgatus<br>**Bartonella**:bacilliformis,grahamii,henselae,quintana,tribocorum<br>**Bifidobacterium**:adolescentis,animalis,dentium,longum<br>**Bordetella**:avium,bronchiseptica,parapertussis,pertussis,petrii<br>**Borrelia**:afzelii,bavariensis,burgdorferi,duttonii,hermsii,recurrentis,turicatae<br>**Bradyrhizobium**:japonicum,sp. BTAi1,sp. ORS278<br>**Brucella**:abortus,canis,melitensis,microti,ovis,suis<br>**Burkholderia**:ambifaria,cenocepacia,glumae,mallei,multivorans,phymatum,phytofirmans, pseudomallei,sp. 383,thailandensis,vietnamiensis,xenovorans<br>**Campylobacter**:concisus,curvus,fetus,hominis,jejuni,lari<br>**Candidatus Phytoplasma**:australiense,mali<br>**Chlamydophila**:abortus,caviae,felis,pneumoniae<br>**Chlorobium**:chlorochromatii,limicola,phaeobacteroides,phaeovibrioides<br>**Chloroflexus**:aggregans,aurantiacus,sp. Y-400-fl<br>**Clostridium**:acetobutylicum,beijerinckii,botulinum,cellulolyticum,difficile,kluyveri,novyi, perfringens,phytofermentans,tetani,thermocellum<br>**Corynebacterium**:aurimucosum,diphtheriae,efficiens,glutamicum,jeikeium,kroppenstedtii, urealyticum<br>**Cupriavidus**:metallidurans,necator,pinatubonensis,taiwanensis<br>**Deinococcus**:deserti,geothermalis,radiodurans<br>**Desulfovibrio**:desulfuricans,magneticus,salexigens,vulgaris<br>**Ehrlichia**:canis,chaffeensis,ruminantium<br>**Erwinia**:amylovora,pyrifoliae,tasmaniensis<br>**Francisella**:novicida,philomiragia,tularensis<br>**Frankia**:alni,sp. CcI3,sp. EAN1pec<br>**Geobacillus**:kaustophilus,sp. WCH70,sp. Y412MC10,sp. Y412MC61,thermodenitrificans<br>**Geobacter**:bemidjiensis,lovleyi,metallireducens,sp. FRC-32,sp. M21, sulfurreducens,uraniireducens<br>**Haemophilus**:ducreyi,influenzae,parasuis<br>**Helicobacter**:acinonychis,hepaticus,mustelae,pylori<br>**Lactobacillus**:acidophilus,brevis,casei,delbrueckii,fermentum,gasseri,helveticus,johnsonii, plantarum,reuteri,rhamnosus,sakei,salivarius<br>**Leptospira**:biflexa,borgpetersenii,interrogans<br>**Listeria**:innocua,monocytogenes,seeligeri,welshimeri<br>**Methanocaldococcus**:fervens,jannaschii,sp. FS406-22,vulcanius<br>**Methanococcus**:aeolicus,maripaludis,vannielii<br>**Methanosarcina**:acetivorans,barkeri,mazei<br>**Methylobacterium**:chloromethanicum,extorquens,nodulans,populi,radiotolerans,sp. 4-46<br>**Mycobacterium**:abscessus,avium,bovis,gilvum,leprae,marinum,smegmatis,sp. JLS,sp. KMS,	

	sp. MCS,tuberculosis,ulcerans,vanbaalenii<br>**Mycoplasma**:agalactiae,arthritidis,capricolum,conjunctivae,crocodyli,gallisepticum, genitalium,hominis,hyopneumoniae,mobile,mycoides,penetrans,pneumoniae,pulmonis, synoviae<br>**Onion yellows phytoplasma**<br>**Pectobacterium**:atrosepticum,carotovorum,wasabiae<br>**Pseudomonas**:aeruginosa,entomophila,fluorescens,mendocina,putida,savastanoi,stutzeri, syringae,syringae group genomosp. 3<br>**Psychrobacter**:arcticus,cryohalolentis,sp. PRwf-1<br>**Pyrobaculum**:aerophilum,arsenaticum,calidifontis,islandicum<br>**Pyrococcus**:abyssi,furiosus,horikoshii<br>**Rhizobium**:etli,leguminosarum,sp. NGR234<br>**Rhodococcus**:erythropolis,jostii,opacus<br>**Rickettsia**:africae,akari,bellii,canadensis,conorii,felis,massiliae,peacockii,prowazekii, rickettsii,typhi<br>**Shewanella**:amazonensis,baltica,denitrificans,frigidimarina,halifaxensis,loihica,oneidensis, pealeana,piezotolerans,putrefaciens,sediminis,sp. ANA-3,sp. MR-4,sp. MR-7, sp. W3-18-1,violacea,woodyi<br>**Shigella**:boydii,dysenteriae,flexneri,sonnei<br>**Staphylococcus**:aureus,carnosus,epidermidis,haemolyticus,lugdunensis,saprophyticus<br>**Streptococcus**:agalactiae,dysgalactiae,equi,gallolyticus,gordonii,mitis,mutans,pneumoniae, pyogenes,sanguinis,suis,thermophilus,uberis<br>**Streptomyces**:avermitilis,coelicolor,griseus,scabiei<br>**Sulfolobus**:acidocaldarius,islandicus,solfataricus,tokodaii<br>**Thermoanaerobacter**:italicus,pseudethanolicus,sp. X514,tengcongensis<br>**Thermococcus**:gammatolerans,kodakarensis,onnurineus,sibiricus<br>**Thermotoga**:lettingae,maritima,naphthophila,neapolitana,petrophila,sp. RQ2<br>**Vibrio**:cholerae,harveyi,parahaemolyticus,sp. Ex25,splendidus,vulnificus<br>**Wolbachia**:endosymbiont of Brugia malayi,endosymbiont of Culex quinquefasciatus,endosymbiont of Drosophila melanogaster,sp. wRi<br>**Xanthomonas**:albilineans,axonopodis,campestris,euvesicatoria,oryzae<br>**Yersinia**:enterocolitica,pestis,pseudotuberculosis	
Genus	14: Shigella; Cupriavidus; Rickettsia; Bacillus; Pectobacterium; Yersinia; Ehrlichia; Anaplasma; Geobacillus; Erwinia; Thermococcus; Burkholderia; Wolbachia; Pyrococcus	47: Vibrio; Rhodococcus; Sulfolobus; Methanosarcina; Chlorobium; Brucella; Geobacter; Listeria; Shewanella; Pseudomonas; Streptomyces; Bifidobacterium; Pyrobaculum; Clostridium; Chloroflexus; Helicobacter; Xanthomonas;Methanocaldococcus; Francisella; Bacteroides; Campylobacter; Chlamydophila; Borrelia; Arthrobacter; Staphylococcus; Methanococcus; Streptococcus; Thermotoga; Psychrobacter; Candidatus Phytoplasma; Bradyrhizobium; Bartonella; Corynebacterium; Thermoanaerobacter; Leptospira; Frankia; Lactobacillus; Acidovorax; Mycobacterium; Anaeromyxobacter; Desulfovibrio; Deinococcus; Mycoplasma; Rhizobium; Bordetella; Haemophilus; Methylobacterium
Family	29: Streptococcaceae; Frankiaceae; Listeriaceae; Rickettsiaceae; Bradyrhizobiaceae; Nocardiaceae; Methanocaldococcaceae; Pseudomonadaceae; Burkholderiaceae; Staphylococcaceae; Corynebacteriaceae; Methylobacteriaceae; Anaplasmataceae; Lactobacillaceae; Rhizobiaceae; Spirochaetaceae; Mycobacteriaceae; Bartonellaceae; Micrococcaceae; Brucellaceae; Bacillaceae; Moraxellaceae; Campylobacteraceae; Helicobacteraceae; Streptomycetaceae; Comamonadaceae; Alcaligenaceae; Leptospiraceae; Methanococcaceae	24: Thermotogaceae; Shewanellaceae; Methanosarcinaceae; Mycoplasmataceae; Geobacteraceae; Clostridiaceae; Myxococcaceae; Thermoproteaceae; Desulfovibrionaceae;

		Thermococcaceae; Chloroflexaceae; Acholeplasmataceae; Pasteurellaceae; Francisellaceae; Chlorobiaceae; Chlamydiaceae; Xanthomonadaceae; Sulfolobaceae; Bacteroidaceae; Enterobacteriaceae; Bifidobacteriaceae; Thermoanaerobacteraceae; Vibrionaceae; Deinococcaceae
Order	22: Thermoanaerobacterales; Sulfolobales; Rickettsiales; Actinomycetales; Pasteurellales; Clostridiales; Bifidobacteriales; Thiotrichales; Bacillales; Thermoproteales; Lactobacillales; Myxococcales; Mycoplasmatales; Alteromonadales; Rhizobiales; Vibrionales; Enterobacteriales; Desulfuromonadales; Xanthomonadales; Pseudomonadales; Desulfovibrionales; Acholeplasmatales	12: Thermococcales; Spirochaetales; Chlorobiales; Chlamydiales; Thermotogales; Methanosarcinales; Burkholderiales; Deinococcales; Chloroflexales; Campylobacterales; Methanococcales; Bacteroidales
Class	10: Betaproteobacteria; Thermococci; Gammaproteobacteria; Methanococci; Epsilonproteobacteria; Bacilli; Methanomicrobia; Clostridia; Alphaproteobacteria; Deltaproteobacteria	10: Chlorobia; Chloroflexi class; Bacteroidia; Deinococci; Actinobacteria class; Thermotogae class; Chlamydiae class; Mollicutes; Thermoprotei; Spirochaetes class
Phylum	13: Spirochaetes; Tenericutes; Chlamydiae; Firmicutes; Bacteroidetes; Chloroflexi; Actinobacteria; Chlorobi; Thermotogae; Deinococcus-Thermus; Proteobacteria; Euryarchaeota; Crenarchaeota	
