# Supplementary material for: Classifying short genomic fragments from novel lineages using composition and homology
Source: BMC Bioinformatics. 2011 Aug 9;12:328. doi: 10.1186/1471-2105-12-328 (PMC3173459; doi:10.1186/1471-2105-12-328)
Supplement: Additional file 2 — Impact of n-mer length on NB performance. Classification performance of NB classifiers with models built from oligonucleotides of varying length n and strain-level (Additional file 2, Table S2), species-level (Additional file 2, Table S3), genus-level (Additional file 2, Table S4), and family-level (Additional file 2, Table S5) lineages removed from the training set. The average sensitivity (Sn), false negative rate (FNr), and specificity (Sp) are reported for the 200 bp test set. [file 1471-2105-12-328-S2.PDF]

Additional File 2

Classifying short genomic fragments from novel lineages using composition and homology

Donovan H. Parks[1,§], Norman J. MacDonald[1,§], and Robert G. Beiko[1,*]

[1]Faculty of Computer Science, Dalhousie University, 6050 University Avenue, Halifax, Nova Scotia, Canada B3H 1W5

§ These authors contributed equally to this work.
[*] To whom correspondence should be addressed (beiko@cs.dal.ca).

Table S2. Classification performance of NB classifiers with models built from oligonucleotides of varying length *n* and strain-level lineages removed from the training set. In Tables S2-S5, the average sensitivity (*Sn*), false negative rate (*FNr*), and specificity (*Sp*) are reported for the 200 bp test set, with best scores for each taxonomic rank indicated in bold.

	**Species**			**Genus**			**Family**			**Order**			**Class**			**Phylum**			**Domain**		
n	*Sn*	*FNr*	*Sp*	*Sn*	*FNr*	*Sp*	*Sn*	*FNr*	*Sp*	*Sn*	*FNr*	*Sp*	*Sn*	*FNr*	*Sp*	*Sn*	*FNr*	*Sp*	*Sn*	*FNr*	*Sp*
**3**	13	87	33	32	68	38	33	67	39	37	63	40	44	56	41	42	58	40	70	30	59
**4**	17	83	39	39	62	45	40	60	46	43	57	46	50	50	47	48	52	45	74	26	62
**5**	20	80	43	43	57	50	45	55	50	48	52	51	55	45	52	52	48	49	77	23	64
**6**	22	78	47	48	52	54	49	51	54	53	47	55	58	42	55	56	44	52	81	19	66
**7**	26	74	51	52	48	58	53	47	58	57	43	58	62	38	59	60	40	55	83	17	68
**8**	31	69	57	56	44	62	58	42	62	61	39	62	66	34	63	64	37	59	85	15	70
**9**	40	60	64	64	36	69	65	35	69	67	33	69	71	29	69	70	30	68	88	12	75
**10**	57	43	76	76	24	81	76	24	80	77	23	80	79	21	79	78	22	79	90	10	83
**11**	68	32	83	83	17	92	83	17	91	83	17	90	82	18	89	80	20	92	90	10	94
**12**	70	30	85	84	16	94	83	17	93	83	17	92	80	20	91	78	22	94	85	15	96
**13**	74	26	86	88	12	**95**	87	13	**95**	86	14	93	83	17	**93**	82	18	**95**	89	11	97
**14**	77	23	86	90	10	**95**	**90**	**10**	**95**	89	11	**94**	86	14	**93**	86	14	**95**	94	6	**98**
**15**	**78**	**22**	**87**	**91**	**9**	**95**	**90**	**10**	**95**	**90**	**10**	**94**	**88**	**12**	**93**	**87**	**12**	93	**96**	**4**	97

Table S3. Classification performance of NB classifiers with models built from oligonucleotides of varying length and species-level lineages removed from the training set.

	Species			Genus			Family			Order			Class			Phylum			Domain		
n	*Sn*	*FNr*	*Sp*	*Sn*	*FNr*	*Sp*	*Sn*	*FNr*	*Sp*	*Sn*	*FNr*	*Sp*	*Sn*	*FNr*	*Sp*	*Sn*	*FNr*	*Sp*	*Sn*	*FNr*	*Sp*
**3**	-	-	-	26	74	26	28	72	28	32	68	31	37	63	35	39	61	36	73	27	70
**4**	-	-	-	31	69	31	33	67	33	37	63	36	42	58	40	43	57	41	75	25	73
**5**	-	-	-	35	65	35	37	63	37	41	59	40	46	54	44	47	53	45	77	23	75
**6**	-	-	-	39	61	39	40	60	40	44	56	43	49	51	47	50	50	48	79	21	77
**7**	-	-	-	41	59	42	43	57	43	47	53	46	51	49	50	52	48	51	80	20	78
**8**	-	-	-	44	56	44	45	55	45	49	51	48	53	47	53	54	46	53	81	19	79
**9**	-	-	-	46	54	47	48	52	48	51	49	51	55	45	55	**56**	**44**	56	**82**	**18**	81
**10**	-	-	-	**49**	**51**	51	**50**	**50**	51	**53**	**47**	55	**56**	**44**	57	**56**	**44**	59	**82**	**18**	82
**11**	-	-	-	48	52	55	48	52	54	49	51	58	51	49	58	49	51	61	76	24	84
**12**	-	-	-	43	57	58	42	58	57	44	57	60	45	55	60	43	57	63	69	31	84
**13**	-	-	-	42	58	61	41	59	61	42	58	64	43	57	63	42	58	66	64	36	85
**14**	-	-	-	45	55	**64**	44	56	**63**	44	56	**67**	45	55	**65**	44	56	**68**	65	35	**86**
**15**	-	-	-	46	54	63	46	54	62	45	55	66	45	55	**65**	48	52	67	68	32	85

Table S4. Classification performance of NB classifiers with models built from oligonucleotides of varying length and genus-level lineages removed from the training set.

	Species			Genus			Family			Order			Class			Phylum			Domain		
n	*Sn*	*FNr*	*Sp*	*Sn*	*FNr*	*Sp*	*Sn*	*FNr*	*Sp*	*Sn*	*FNr*	*Sp*	*Sn*	*FNr*	*Sp*	*Sn*	*FNr*	*Sp*	*Sn*	*FNr*	*Sp*
**3**	-	-	-	-	-	-	13	87	56	20	80	71	27	73	56	40	60	55	70	30	70
**4**	-	-	-	-	-	-	15	85	59	24	76	75	30	70	60	43	57	59	71	29	71
**5**	-	-	-	-	-	-	17	83	60	26	74	77	33	67	62	46	54	62	72	28	73
**6**	-	-	-	-	-	-	18	82	60	28	72	79	35	65	63	48	52	63	73	27	73
**7**	-	-	-	-	-	-	19	81	61	29	71	80	37	63	64	49	51	64	73	27	74
**8**	-	-	-	-	-	-	**20**	**80**	**62**	**31**	**69**	81	**38**	**62**	65	**51**	**49**	**66**	**74**	**26**	75
**9**	-	-	-	-	-	-	**20**	**80**	**62**	**31**	**69**	**82**	**38**	**62**	**66**	**51**	**49**	65	**74**	**26**	75
**10**	-	-	-	-	-	-	19	81	**62**	29	71	81	36	64	64	50	50	63	**74**	**26**	75
**11**	-	-	-	-	-	-	18	82	59	27	73	79	33	67	62	50	50	62	71	29	**79**
**12**	-	-	-	-	-	-	14	86	56	21	79	75	27	73	58	46	54	60	65	35	78
**13**	-	-	-	-	-	-	11	89	55	16	84	69	22	78	52	42	58	57	60	40	76
**14**	-	-	-	-	-	-	11	89	49	15	85	66	20	80	49	40	60	54	58	42	74
**15**	-	-	-	-	-	-	11	89	46	16	84	66	21	79	49	38	62	52	57	43	70

Table S5. Classification performance of NB classifiers with models built from oligonucleotides of varying length and family-level lineages removed from the training set.

	**Species**			**Genus**			**Family**			**Order**			**Class**			**Phylum**			**Domain**		
n	*Sn*	*FNr*	*Sp*	*Sn*	*FNr*	*Sp*	*Sn*	*FNr*	*Sp*	*Sn*	*FNr*	*Sp*	*Sn*	*FNr*	*Sp*	*Sn*	*FNr*	*Sp*	*Sn*	*FNr*	*Sp*
**3**	-	-	-	-	-	-	-	-	-	14	86	22	20	80	27	31	69	35	57	43	54
**4**	-	-	-	-	-	-	-	-	-	15	85	24	21	79	29	32	68	37	58	42	55
**5**	-	-	-	-	-	-	-	-	-	16	84	25	22	78	31	33	67	37	58	42	56
**6**	-	-	-	-	-	-	-	-	-	16	84	26	23	77	31	34	66	38	58	42	56
**7**	-	-	-	-	-	-	-	-	-	17	83	27	24	76	**33**	34	66	**39**	**59**	**41**	**57**
**8**	-	-	-	-	-	-	-	-	-	17	83	28	24	76	**33**	35	65	**39**	**59**	**41**	56
**9**	-	-	-	-	-	-	-	-	-	**18**	**82**	28	**25**	**75**	**33**	**36**	**64**	**39**	58	42	56
**10**	-	-	-	-	-	-	-	-	-	**18**	**82**	**29**	**25**	**75**	**33**	**36**	**64**	**39**	55	45	54
**11**	-	-	-	-	-	-	-	-	-	**18**	**82**	28	24	76	32	**36**	**64**	37	51	49	52
**12**	-	-	-	-	-	-	-	-	-	17	83	27	22	78	31	**36**	**64**	37	51	49	51
**13**	-	-	-	-	-	-	-	-	-	15	85	25	19	81	29	37	63	35	50	50	51
**14**	-	-	-	-	-	-	-	-	-	14	86	26	18	82	29	36	64	36	51	49	53
**15**	-	-	-	-	-	-	-	-	-	14	86	24	18	82	27	35	65	35	52	48	54
