# Supplementary material for: Classifying short genomic fragments from novel lineages using composition and homology
Source: BMC Bioinformatics. 2011 Aug 9;12:328. doi: 10.1186/1471-2105-12-328 (PMC3173459; doi:10.1186/1471-2105-12-328)
Supplement: Additional file 3 — Performance of rank-specific classifiers. Average classification performance of rank-specific classifiers on 200 bp (Additional file 3, Figure S1), 400 bp (Additional file 3, Figure S2), and 1000 bp (Additional file 3, Figure S3) query fragments using a leave-one-out evaluation framework. Fragments were considered correctly classified if they were assigned to the species (S), genus (G), family (F), order (O), class (C), phylum (P), or domain (D) of their source genome. Corresponding results for absolute measures of performance are given in Additional file 3, Figures S4-S6. [file 1471-2105-12-328-S3.PDF]

Additional File 3

Classifying short genomic fragments from novel lineages using composition and homology

Donovan H. Parks[1,§], Norman J. MacDonald[1,§], and Robert G. Beiko[1,*]

[1]Faculty of Computer Science, Dalhousie University, 6050 University Avenue, Halifax, Nova Scotia, Canada B3H 1W5

§ These authors contributed equally to this work.
[*] To whom correspondence should be addressed (beiko@cs.dal.ca).

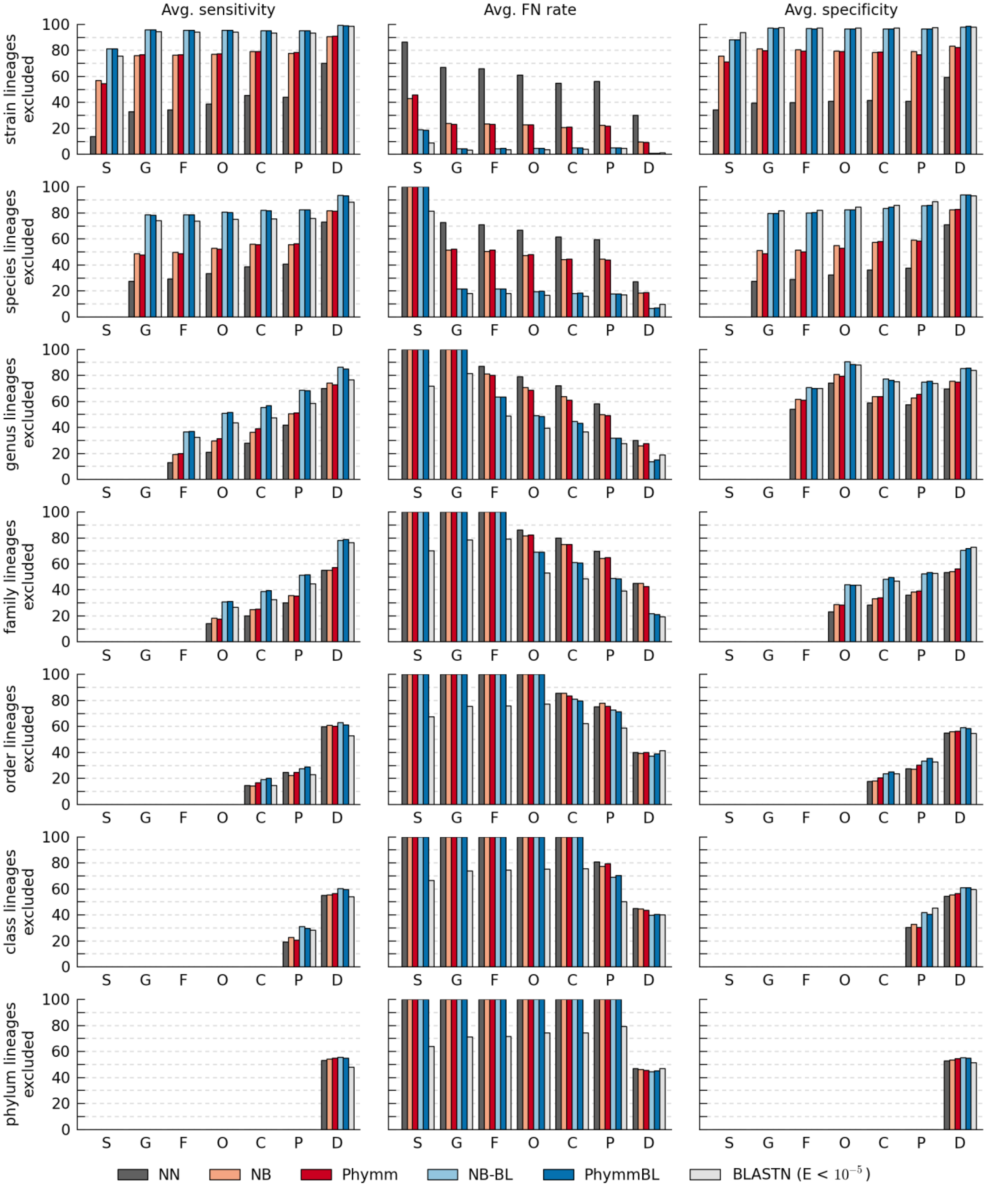

Figure S1. Average classification performance of rank-specific classifiers on 200 bp query fragments using a leave-one-out evaluation framework. Fragments were considered correctly classified if they were assigned to the species (S), genus (G), family (F), order (O), class (C), phylum (P), or domain (D) of their source genome.

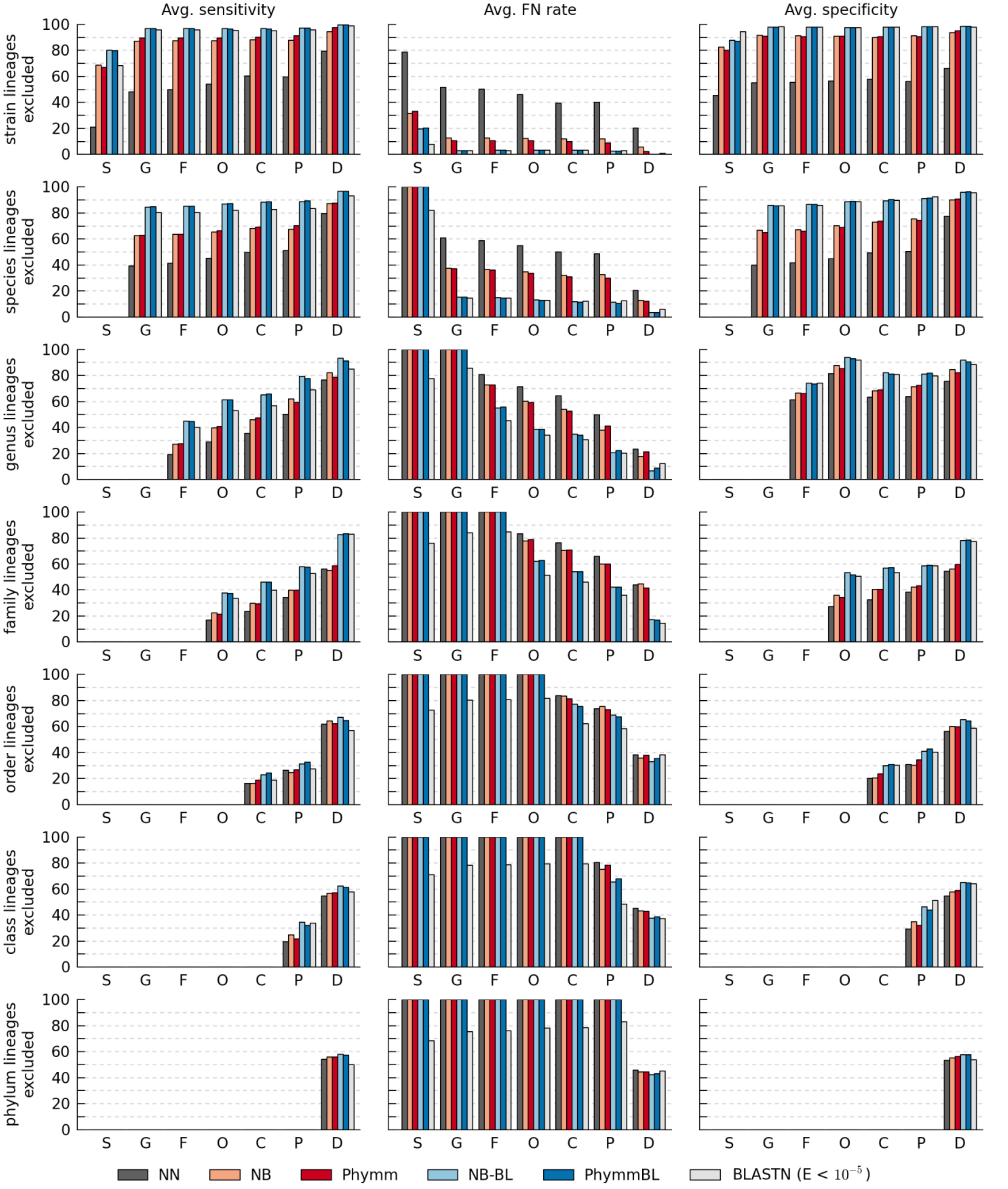

Figure S2. Average classification performance of rank-specific classifiers on 400 bp query fragments using a leave-one-out evaluation framework. Fragments were considered correctly classified if they were assigned to the species (S), genus (G), family (F), order (O), class (C), phylum (P), or domain (D) of their source genome.

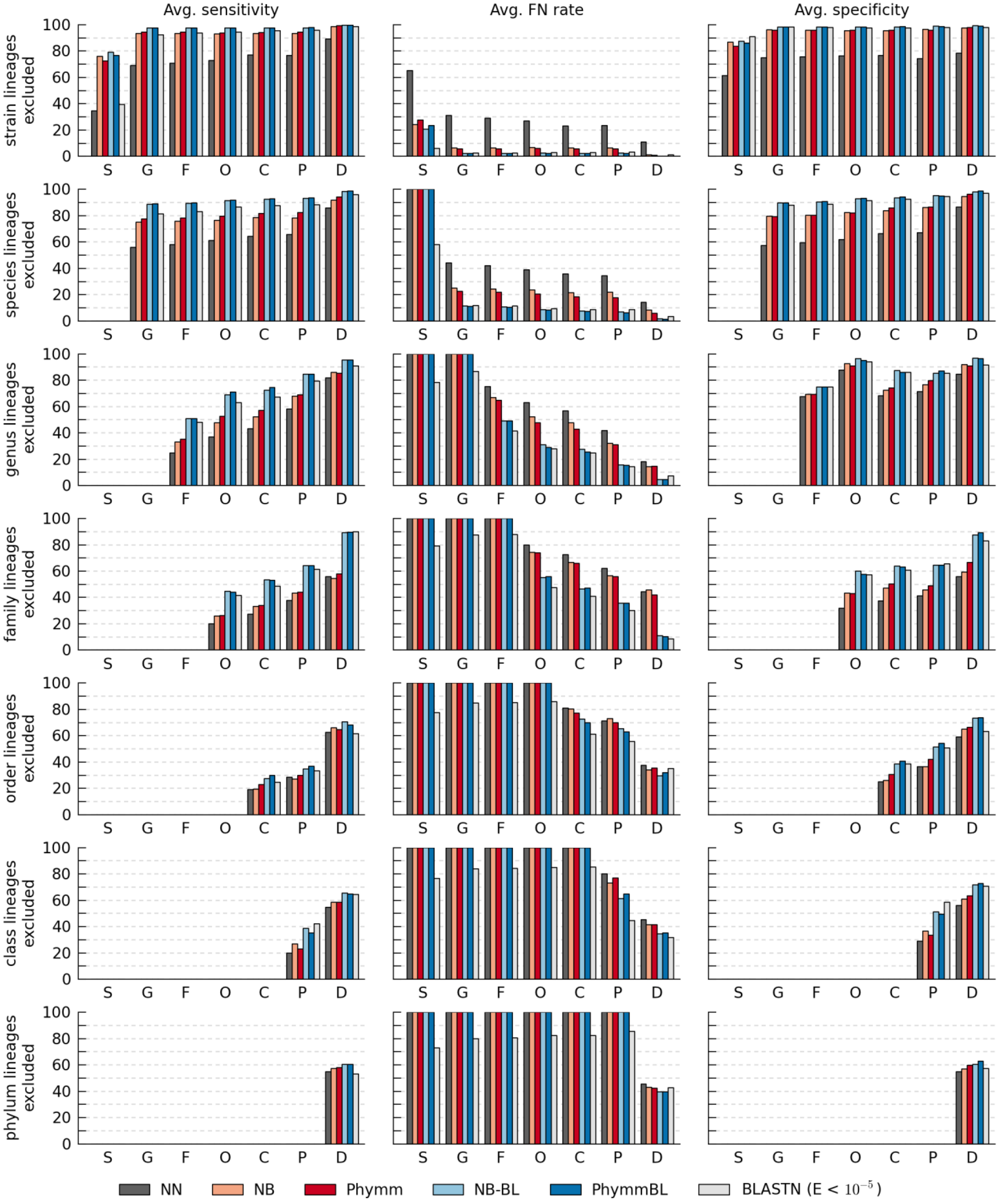

Figure S3. Average classification performance of rank-specific classifiers on 1000 bp query fragments using a leave-one-out evaluation framework. Fragments were considered correctly classified if they were assigned to the species (S), genus (G), family (F), order (O), class (C), phylum (P), or domain (D) of their source genome.

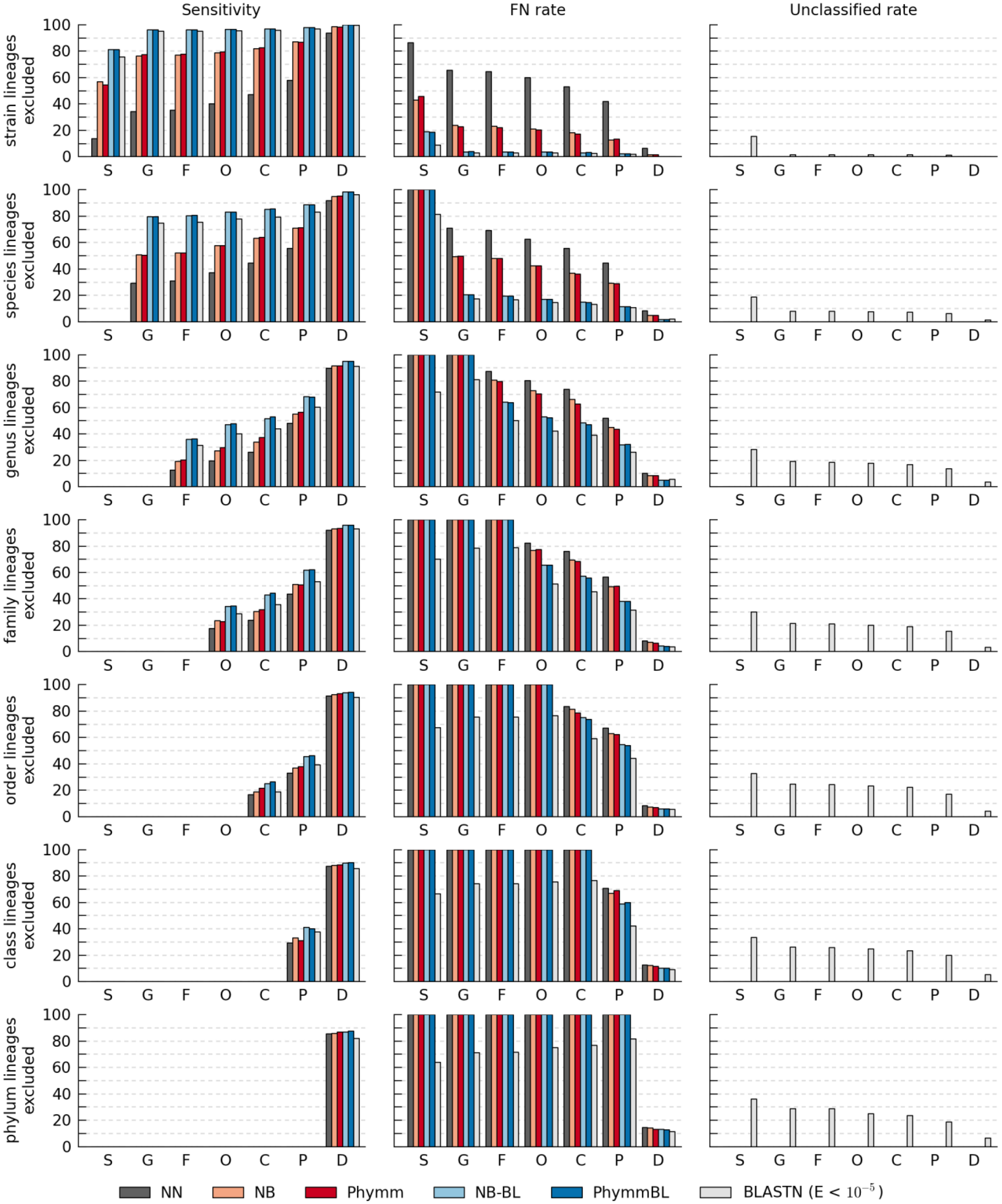

Figure S4. Absolute classification performance of rank-specific classifiers over all 200 bp query fragments using a leave-one-out evaluation framework. Fragments were considered correctly classified if they were assigned to the species (S), genus (G), family (F), order (O), class (C), phylum (P), or domain (D) of their source genome.

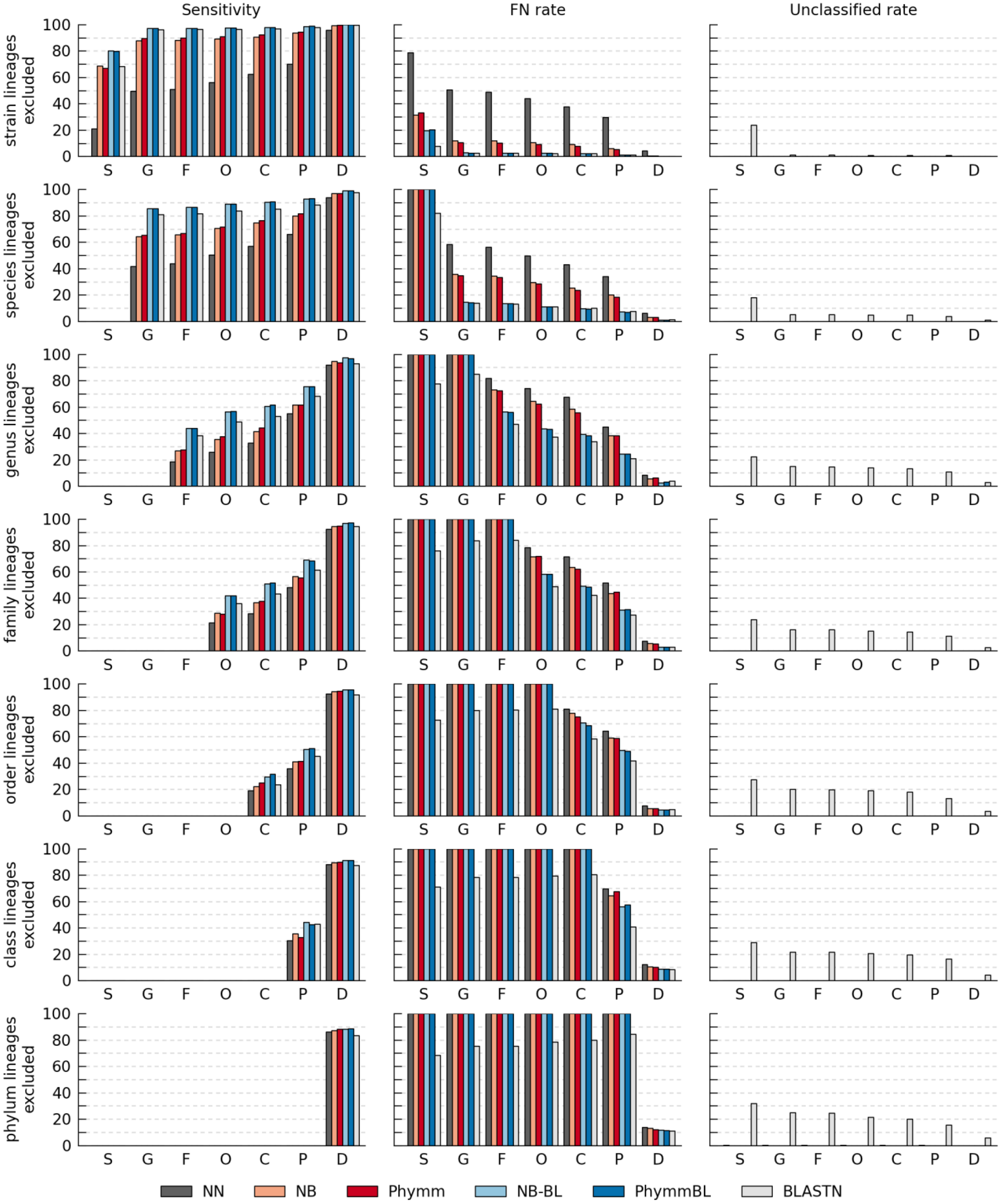

Figure S5. Absolute classification performance of rank-specific classifiers over all 400 bp query fragments using a leave-one-out evaluation framework. Fragments were considered correctly classified if they were assigned to the species (S), genus (G), family (F), order (O), class (C), phylum (P), or domain (D) of their source genome.

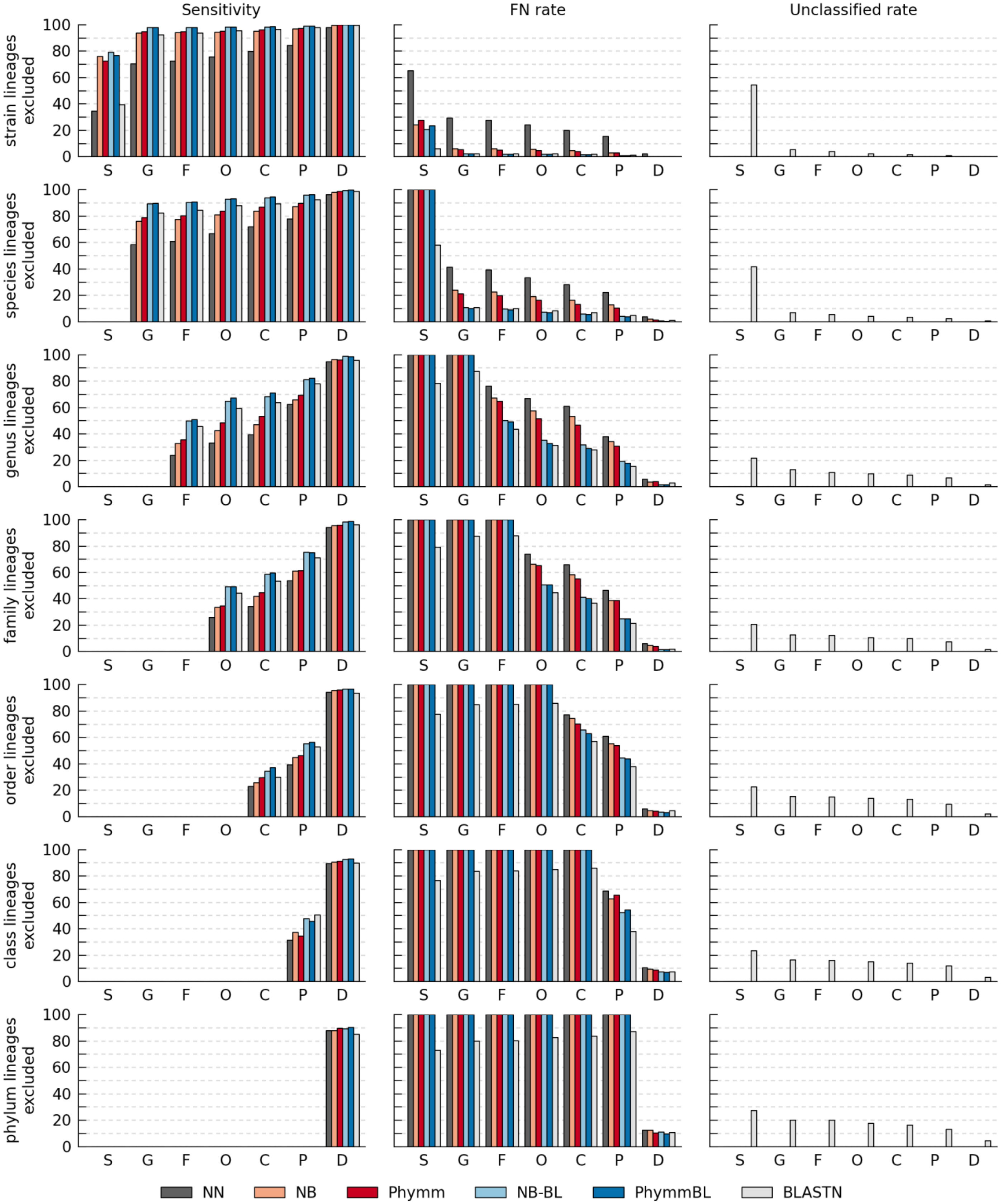

Figure S6. Absolute classification performance of rank-specific classifiers over all 1000 bp query fragments using a leave-one-out evaluation framework. Fragments were considered correctly classified if they were assigned to the species (S), genus (G), family (F), order (O), class (C), phylum (P), or domain (D) of their source genome.
