# Supplementary material for: Classifying short genomic fragments from novel lineages using composition and homology
Source: BMC Bioinformatics. 2011 Aug 9;12:328. doi: 10.1186/1471-2105-12-328 (PMC3173459; doi:10.1186/1471-2105-12-328)
Supplement: Additional file 4 — Classification results on specific taxonomic groups. Classification results for NB, Phymm, and BLASTN on specific taxonomic groups detailed as a confusion matrix at the phylum level (Additional file 4, Table S6) and a summary table at the genus level (Additional file 4, Table S7). Results are reported for 200 bp query fragments with species-level lineages excluded from the training set. [file 1471-2105-12-328-S4.PDF]

Additional File 4

Classifying short genomic fragments from novel lineages using composition and homology

Donovan H. Parks[1,§], Norman J. MacDonald[1,§], and Robert G. Beiko[1,*]

[1]Faculty of Computer Science, Dalhousie University, 6050 University Avenue, Halifax, Nova Scotia, Canada B3H 1W5

§ These authors contributed equally to this work.

[*] To whom correspondence should be addressed (beiko@cs.dal.ca).

Table S6. Confusion matrix of phylum-level classifications on 200 bp query fragments with species-level lineages excluded from the training set. Results are reported for the NB, Phymm, and BLASTN classifiers. The total number of fragments classified from each group is indicated in parentheses.

	**Predicted**													
**Actual** (NB / Phymm / BLASTN)	Actinobacteria	Bacteroidetes	Chlamydiae	Chlorobi	Chloroflexi	Crenarchaeota	Deinococcus-Thermus	Euryarchaeota	Firmicutes	Proteobacteria	Spirochaetes	Tenericutes	Thermotogae	Unclassified
Actinobacteria	**2719**	2	4	7	27	5	47	10	52	816	3	5	5	0
(3700)	**2709**	0	6	14	23	6	53	7	60	818	1	3	3	0
	**2940**	2	0	2	0	0	7	6	32	495	3	2	3	208
Bacteroidetes	5	**122**	0	15	3	2	0	11	87	42	5	7	7	0
(300)	7	**99**	3	11	2	5	0	14	84	58	8	7	7	0
	3	**166**	1	1	0	1	0	6	31	42	4	3	0	42
Chlamydiae	1	4	**146**	1	0	10	0	46	102	49	23	7	7	0
(400)	1	1	**177**	1	0	11	0	25	106	60	8	6	6	0
	2	0	**345**	0	0	0	0	2	10	18	3	3	1	16
Chlorobi	7	9	0	**105**	7	1	2	11	67	179	2	9	9	0
(400)	16	6	4	**118**	2	1	1	12	53	175	0	11	11	0
	6	2	0	**242**	1	2	1	4	27	76	5	0	0	34
Chloroflexi	23	1	0	1	**206**	0	1	1	10	56	1	0	0	0
(300)	23	0	0	0	**213**	0	1	0	11	51	1	0	0	0
	5	0	0	1	**274**	0	0	1	1	6	0	0	0	12
Crenarchaeota	8	1	7	0	1	**528**	3	61	117	59	4	4	4	0
(800)	8	0	17	1	0	**510**	0	68	107	70	16	2	2	0
	19	1	0	0	1	**577**	2	13	55	63	1	5	0	63
Deinococcus-Thermus	69	0	0	1	1	1	**72**	0	4	152	0	0	0	0
(300)	64	0	1	2	1	1	**61**	2	2	165	0	1	1	0
	19	0	0	0	0	0	**188**	1	2	71	0	1	0	18
Euryarchaeota	16	10	10	6	0	56	0	**998**	357	114	53	37	37	0
(1700)	17	12	33	12	1	57	0	**985**	302	146	52	29	29	0
	15	3	0	0	1	13	0	**1364**	95	71	9	14	3	112
Firmicutes	57	85	17	56	8	84	4	221	**5123**	909	121	35	35	0
(6900)	59	52	55	59	8	106	2	199	**4899**	1049	150	22	22	0
	40	16	3	17	2	15	5	86	**5678**	411	33	72	5	517
Proteobacteria	725	63	35	129	70	82	94	173	1637	**11763**	117	55	55	0
(15100)	798	52	81	125	48	86	86	140	1414	**11882**	135	38	38	0
	197	12	2	22	2	18	11	75	443	**13527**	42	58	9	683
Spirochaetes	1	6	3	3	0	9	0	47	283	73	**511**	8	8	0
(1000)	1	6	12	2	0	7	0	45	166	79	**577**	8	8	0
	2	2	0	0	1	1	1	4	53	27	**856**	6	1	46
Tenericutes	2	0	1	2	0	11	0	38	663	195	105	**881**	2	0
(1900)	1	0	5	0	0	10	0	50	439	211	121	**1062**	1	0
	4	4	4	2	0	10	0	35	345	109	38	**1076**	3	270
Thermotogae	2	6	0	1	0	1	0	35	39	23	18	3	**472**	0
(600)	1	2	8	4	0	1	1	29	60	31	7	1	**455**	0
	0	0	0	0	0	0	0	1	36	17	2	4	**511**	29

Table S7. Performance of NB, Phymm, and BLASTN classifiers at the genus level on 200 bp query fragments with species-level lineages excluded from the training set.

	NB			Phymm			BLASTN			
	Sn	Sp	FNr	Sn	Sp	FNr	Sn	Sp	FNr	Ur
Acidovorax	47.7	43.6	52.3	45.3	46.1	54.7	69.3	78.5	25.7	5.0
Anaeromyxobacter	85.3	69.8	14.7	82.7	69.3	17.3	84.3	78.6	10.3	5.3
Anaplasma	40.3	61.7	59.7	33.3	33.2	66.7	69.0	98.6	19.3	11.7
Arthrobacter	46.0	32.5	54.0	36.3	33.5	63.7	61.7	84.9	26.0	12.3
Bacillus	49.7	44.8	50.3	48.8	45.3	51.2	72.1	71.0	19.0	8.9
Bacteroides	40.7	39.5	59.3	33.0	43.0	67.0	55.3	79.8	27.0	17.7
Bartonella	37.0	56.2	63.0	41.4	46.8	58.6	92.4	91.7	5.2	2.4
Bifidobacterium	30.3	45.5	69.8	37.3	35.1	62.7	65.0	89.0	24.0	11.0
Bordetella	52.2	54.7	47.8	51.8	50.6	48.2	77.2	82.8	19.0	3.8
Borrelia	51.1	59.2	48.9	64.4	51.7	35.6	96.7	88.7	2.6	0.7
Bradyrhizobium	41.3	40.9	58.7	33.3	44.4	66.7	68.7	70.5	21.0	10.3
Brucella	81.5	87.5	18.5	87.5	80.6	12.5	99.7	97.6	0.2	0.2
Burkholderia	74.3	73.6	25.8	72.3	76.9	27.7	87.0	74.8	9.1	3.9
Campylobacter	61.8	48.9	38.2	53.0	61.0	47.0	63.2	73.9	24.2	12.7
Candidatus_Phytoplasma	41.8	64.0	58.3	45.5	44.6	54.5	73.3	85.7	19.0	7.8
Chlamydophila	36.5	65.5	63.5	44.3	44.0	55.8	86.3	97.2	8.3	5.5
Chlorobium	26.3	32.1	73.8	29.5	33.8	70.5	60.5	84.3	26.8	12.8
Chloroflexus	68.7	63.8	31.3	71.0	71.5	29.0	91.3	97.2	4.3	4.3
Clostridium	66.0	24.5	34.0	47.2	38.2	52.8	55.5	52.1	26.6	17.9
Corynebacterium	25.6	40.4	74.4	31.1	34.9	68.9	52.6	91.8	33.1	14.3
Cupriavidus	34.8	33.1	65.3	31.0	31.5	69.0	71.8	66.0	21.0	7.2
Deinococcus	24.0	32.3	76.0	20.3	29.8	79.7	62.7	87.4	29.3	8.0
Desulfovibrio	12.0	13.5	88.0	9.8	13.1	90.3	35.8	65.6	42.0	22.3
Ehrlichia	32.7	28.0	67.3	41.0	40.3	59.0	68.3	82.3	22.7	9.0
Erwinia	46.0	50.4	54.0	41.0	46.9	59.0	84.0	85.4	11.3	4.7
Francisella	64.7	50.9	35.3	59.3	40.7	40.7	90.0	90.0	7.0	3.0
Frankia	36.3	31.0	63.7	27.3	28.6	72.7	56.0	64.9	34.3	9.7
Geobacillus	50.8	47.7	49.2	50.0	50.6	50.0	66.6	76.6	25.8	7.6
Geobacter	47.1	46.2	52.9	39.3	46.2	60.7	67.6	86.5	23.1	9.3
Haemophilus	25.0	35.7	75.0	26.3	32.4	73.7	51.7	83.3	31.7	16.7
Helicobacter	53.3	61.2	46.8	48.5	77.3	51.5	59.3	79.3	26.8	14.0
Lactobacillus	37.8	49.6	62.2	36.8	41.7	63.2	61.5	86.6	25.1	13.5
Leptospira	48.7	40.8	51.3	40.0	58.8	60.0	59.7	77.5	22.7	17.7
Listeria	49.0	50.3	51.0	52.5	43.3	47.5	93.0	79.5	5.3	1.8
Methanocaldococcus	59.3	56.7	40.8	57.8	64.7	42.3	87.0	79.6	8.5	4.5
Methanococcus	36.0	39.9	64.0	39.0	37.7	61.0	67.0	64.6	24.0	9.0
Methanosarcina	38.0	37.1	62.0	36.7	46.0	63.3	75.3	78.5	15.3	9.3
Methylobacterium	59.3	63.0	40.7	59.8	62.8	40.2	78.7	73.2	14.8	6.5
Mycobacterium	55.8	61.3	44.2	62.9	59.3	37.1	88.3	86.0	9.3	2.4
Mycoplasma	39.4	54.6	60.6	45.7	54.1	54.3	50.4	84.0	30.9	18.7
Pectobacterium	41.3	45.6	58.7	38.0	47.7	62.0	84.3	88.8	11.3	4.3
Pseudomonas	58.1	49.7	41.9	55.2	46.9	44.8	81.9	71.8	13.9	4.2
Psychrobacter	39.0	47.0	61.0	37.7	55.7	62.3	74.7	89.2	16.3	9.0
Pyrobaculum	76.8	88.0	23.3	75.3	89.1	24.8	66.8	97.4	23.5	9.8
Pyrococcus	46.7	49.8	53.3	41.3	45.1	58.7	65.0	75.0	25.0	10.0
Rhizobium	45.3	41.1	54.7	45.7	42.8	54.3	68.7	66.7	22.3	9.0
Rhodococcus	45.0	29.2	55.0	32.7	36.6	67.3	68.0	66.4	23.3	8.7
Rickettsia	62.3	83.6	37.7	72.1	69.6	27.9	96.7	93.7	2.4	0.9
Shewanella	59.0	60.5	41.0	56.4	61.4	43.6	83.5	87.2	10.6	5.9
Shigella	56.5	79.6	43.5	67.0	61.9	33.0	93.5	92.6	5.0	1.5
Staphylococcus	46.8	37.7	53.2	42.3	33.3	57.7	77.7	73.0	16.5	5.8
Streptococcus	47.8	57.2	52.2	55.8	44.0	44.2	78.2	83.6	14.8	6.9
Streptomyces	61.3	48.7	38.8	48.8	49.2	51.2	62.7	63.9	25.5	11.8
Sulfolobus	53.5	48.5	46.5	51.0	44.1	49.0	77.3	85.1	14.5	8.3
Thermoanaerobacter	54.5	56.3	45.5	49.8	49.3	50.2	89.0	83.4	6.3	4.8
Thermococcus	58.3	62.1	41.8	54.0	54.7	46.0	59.3	83.2	30.5	10.3
Thermotoga	78.7	73.6	21.3	75.8	78.0	24.2	85.2	95.3	8.8	6.0
Vibrio	50.5	42.3	49.5	43.5	37.4	56.5	77.0	84.0	17.0	6.0
Wolbachia	37.3	79.3	62.7	40.8	50.5	59.3	91.8	95.1	5.0	3.3
Xanthomonas	48.4	60.8	51.6	49.4	53.6	50.6	84.8	84.0	11.4	3.8
Yersinia	49.3	46.8	50.7	67.0	35.4	33.0	86.3	81.2	9.7	4.0
**Average**	**48.7**	**51.1**	**51.3**	**47.8**	**48.8**	**52.2**	**73.9**	**81.7**	**18.0**	**8.2**
