# Supplementary material for: Classifying short genomic fragments from novel lineages using composition and homology
Source: BMC Bioinformatics. 2011 Aug 9;12:328. doi: 10.1186/1471-2105-12-328 (PMC3173459; doi:10.1186/1471-2105-12-328)
Supplement: Additional file 6 — Performance of rank-flexible classifiers. Average classification performance of rank-flexible classifiers on 200 bp (Additional file 6, Figure S7), 400 bp (Additional file 6, Figure S8), and 1000 bp (Additional file 6, Figure S9) query fragments using a leave-one-out evaluation framework. Fragments were considered correctly classified if they were assigned to the species (S), genus (G), family (F), order (O), class (C), phylum (P), or domain (D) of their source genome. Corresponding results for absolute measures of performance are given in Additional file 6, Figures S10-S12. [file 1471-2105-12-328-S6.PDF]

Additional File 6

Classifying short genomic fragments from novel lineages using composition and homology

Donovan H. Parks[1,§], Norman J. MacDonald[1,§], and Robert G. Beiko[1,*]

[1]Faculty of Computer Science, Dalhousie University, 6050 University Avenue, Halifax, Nova Scotia, Canada B3H 1W5

§ These authors contributed equally to this work.

[*] To whom correspondence should be addressed (beiko@cs.dal.ca).

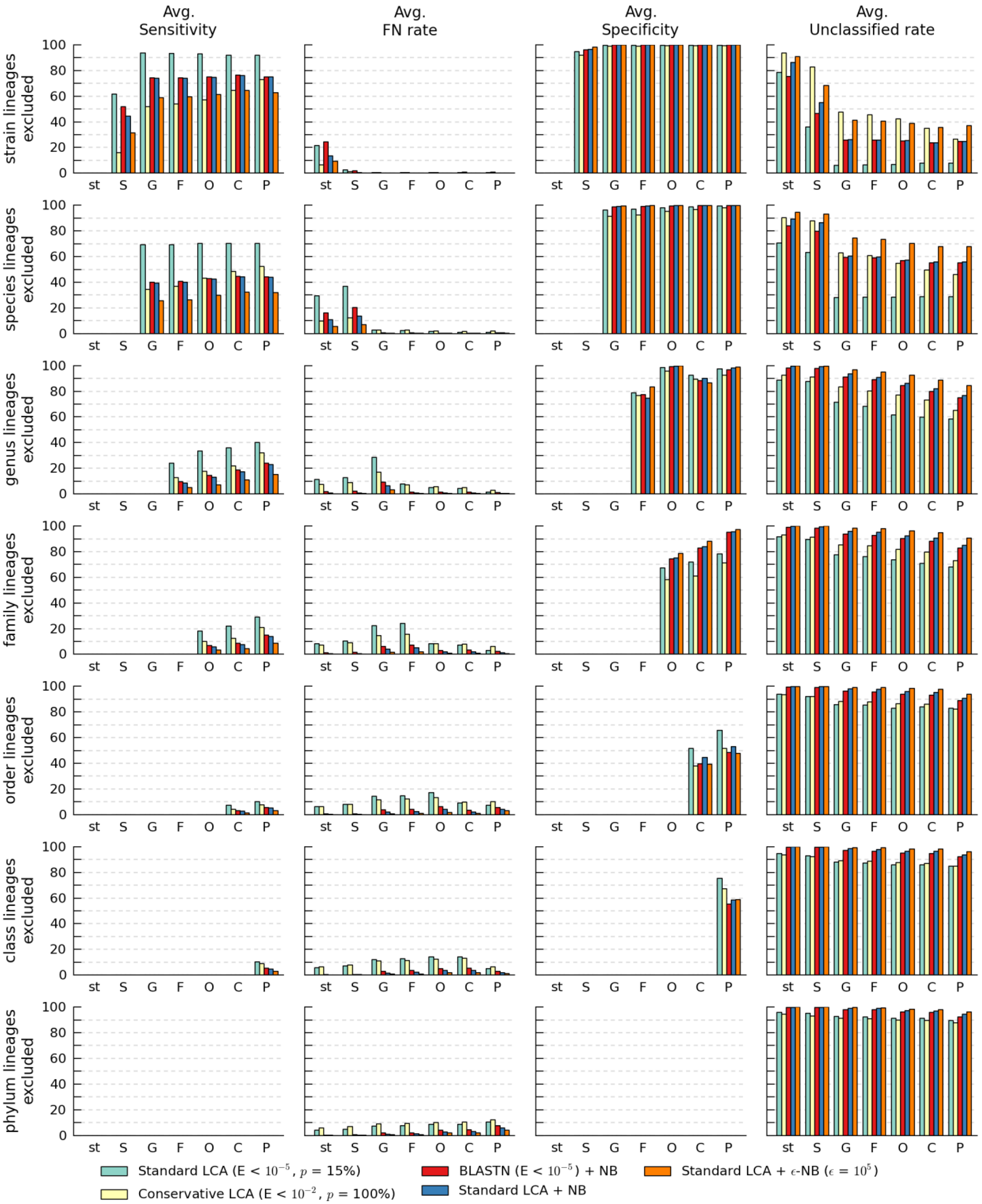

Figure S7. Average classification performance of rank-flexible classifiers on 200 bp query fragments using a leave-one-out evaluation framework. Fragments were considered correctly classified if they were assigned to the strain (st), species (S), genus (G), family (F), order (O), class (C), or phylum (P) of their source genome.

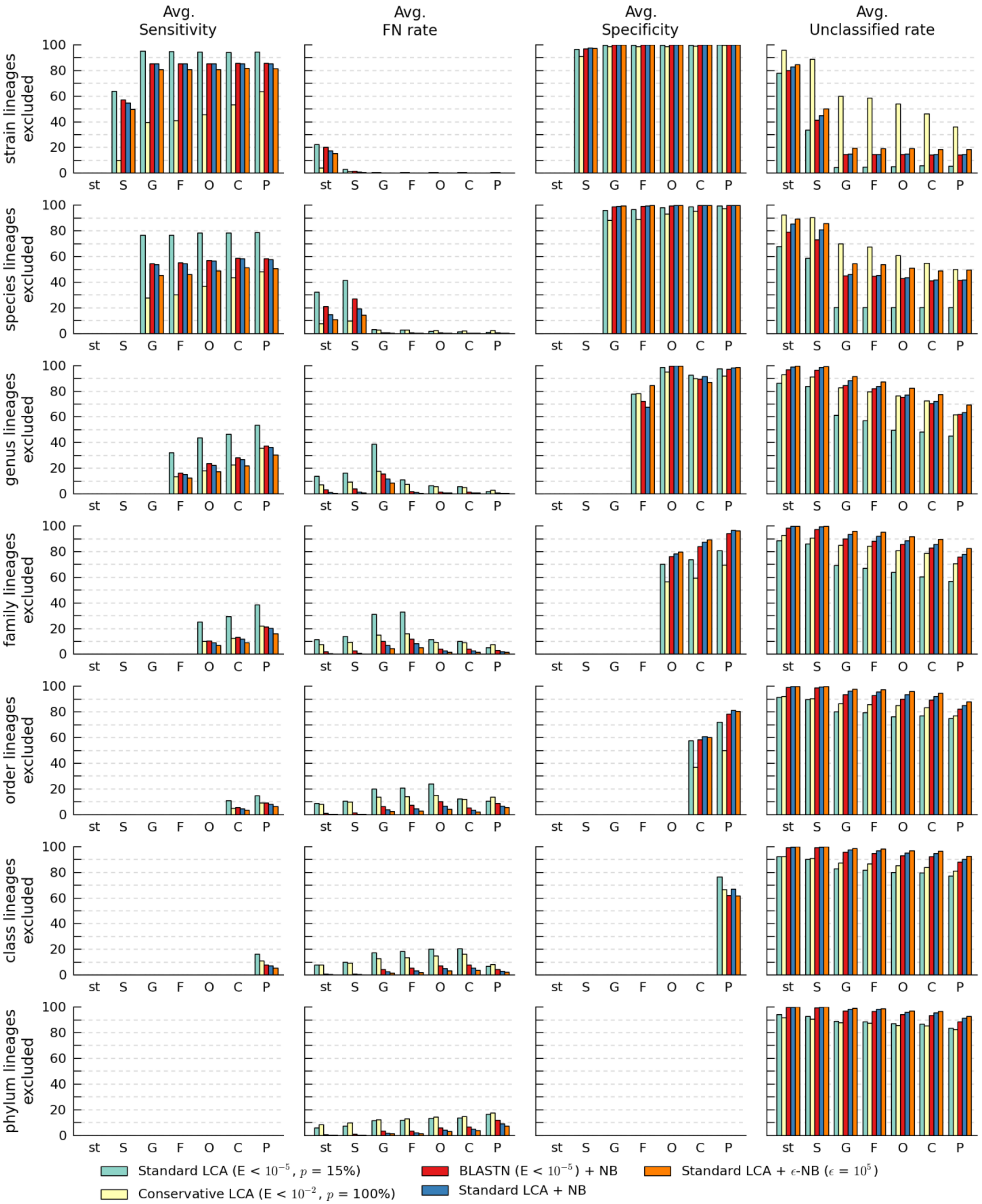

Figure S8. Average classification performance of rank-flexible classifiers on 400 bp query fragments using a leave-one-out evaluation framework. Fragments were considered correctly classified if they were assigned to the strain (st), species (S), genus (G), family (F), order (O), class (C), or phylum (P) of their source genome.

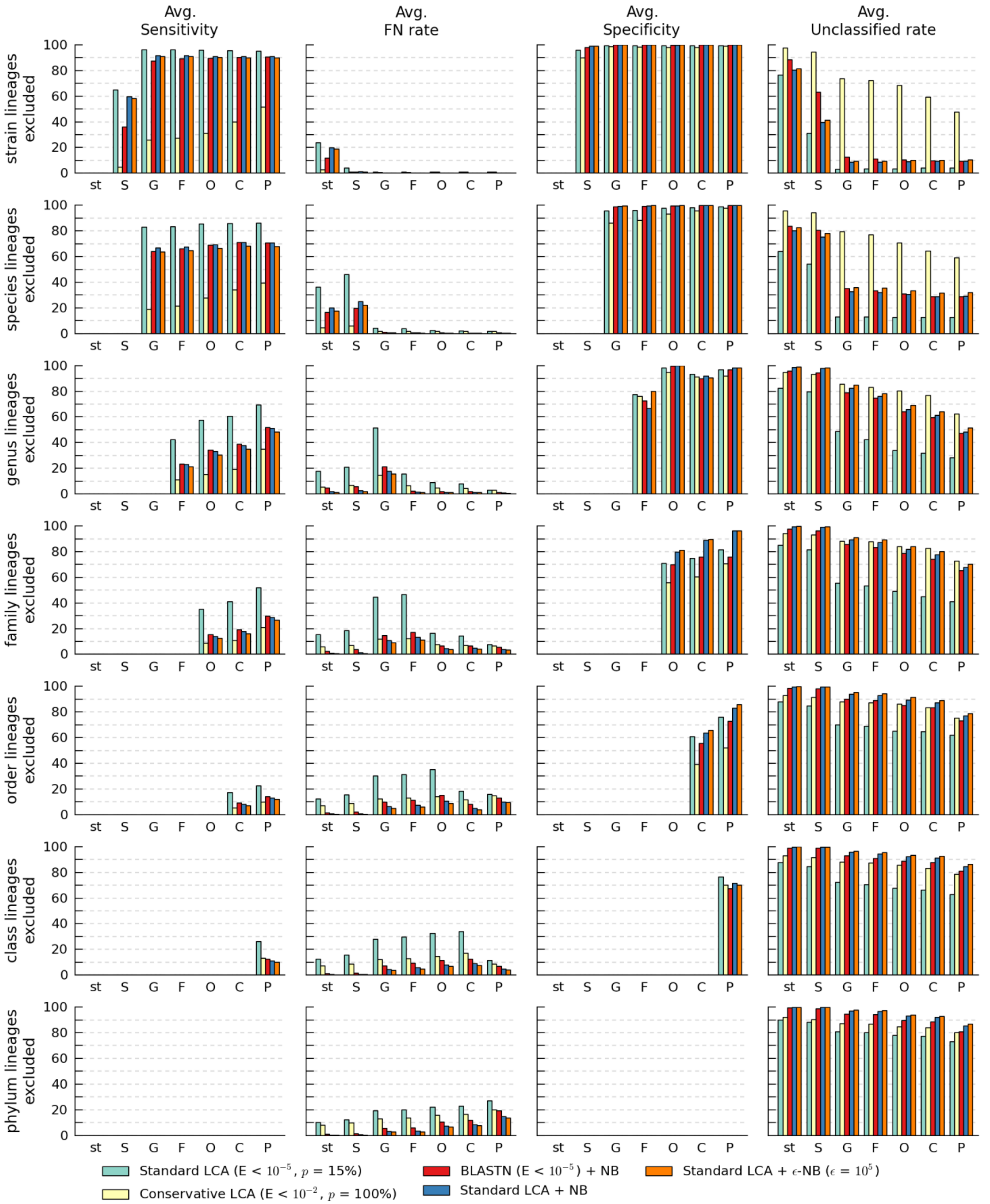

Figure S9. Average classification performance of rank-flexible classifiers on 1000 bp query fragments using a leave-one-out evaluation framework. Fragments were considered correctly classified if they were assigned to the strain (st), species (S), genus (G), family (F), order (O), class (C), or phylum (P) of their source genome.

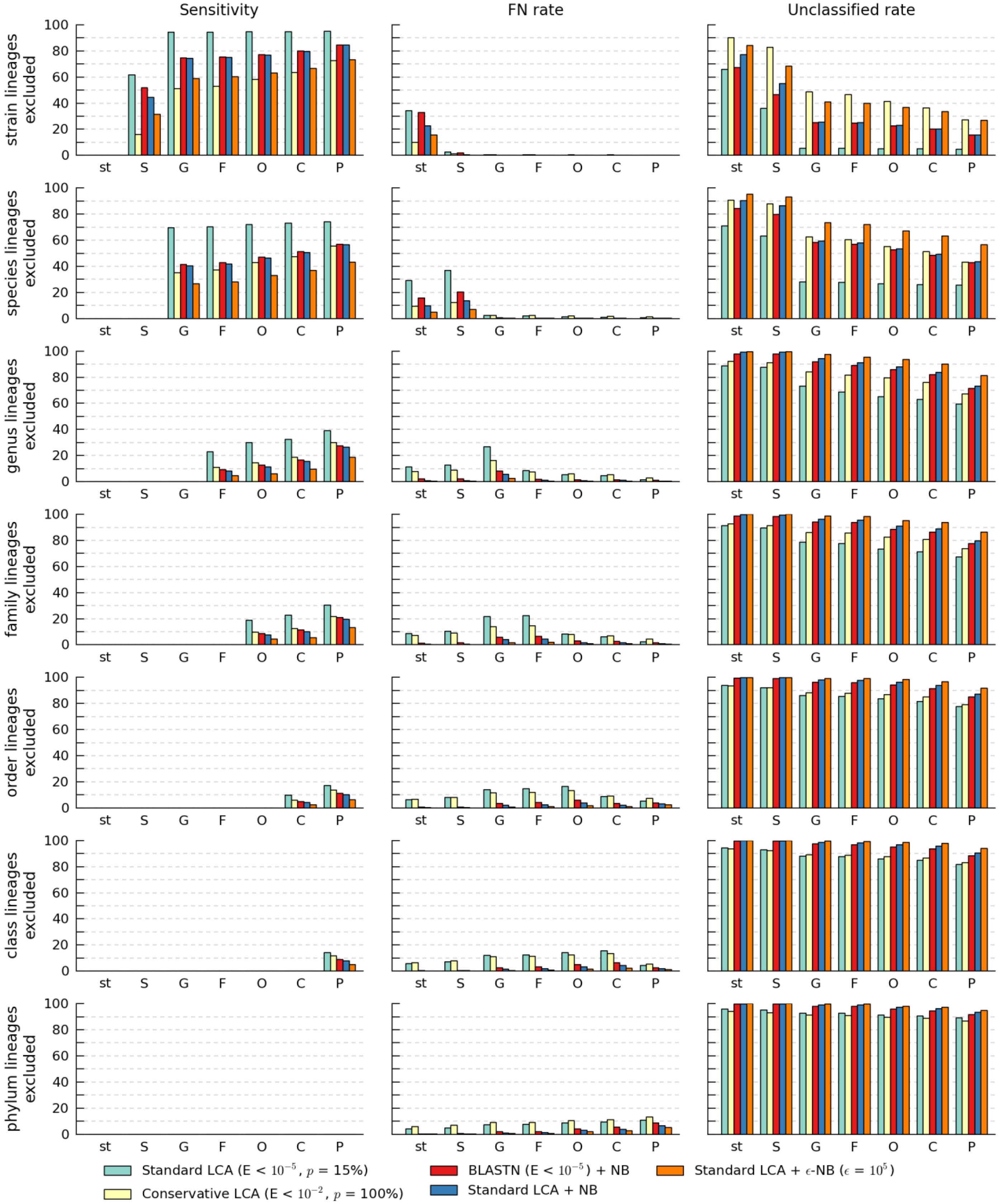

Figure S10. Absolute classification performance of rank-flexible classifiers over all 200 bp query fragments using a leave-one-out evaluation framework. Fragments were considered correctly classified if they were assigned to the strain (st), species (S), genus (G), family (F), order (O), class (C), or phylum (P) of their source genome.

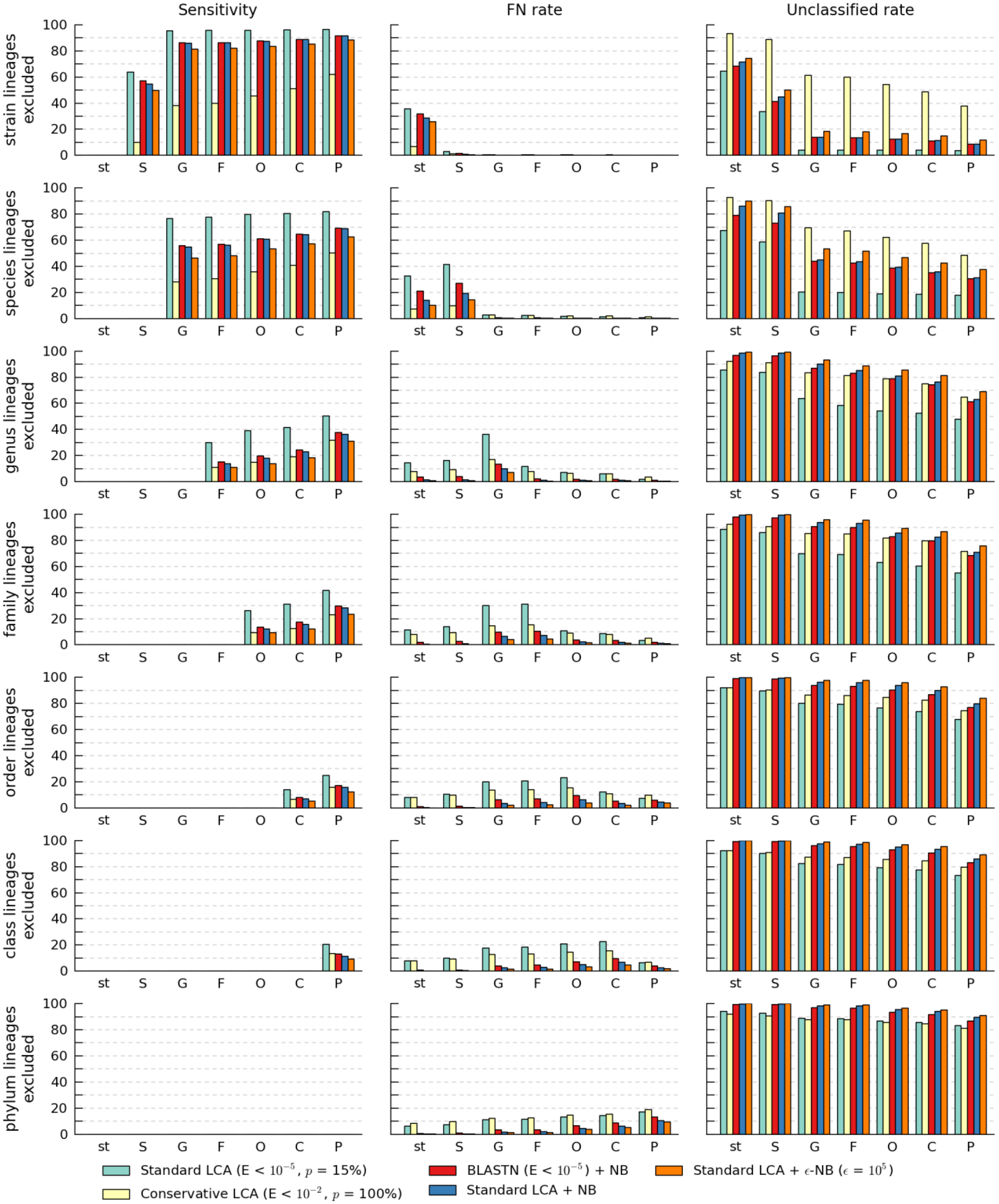

Figure S11. Absolute classification performance of rank-flexible classifiers over all 400 bp query fragments using a leave-one-out evaluation framework. Fragments were considered correctly classified if they were assigned to the strain (st), species (S), genus (G), family (F), order (O), class (C), or phylum (P) of their source genome.

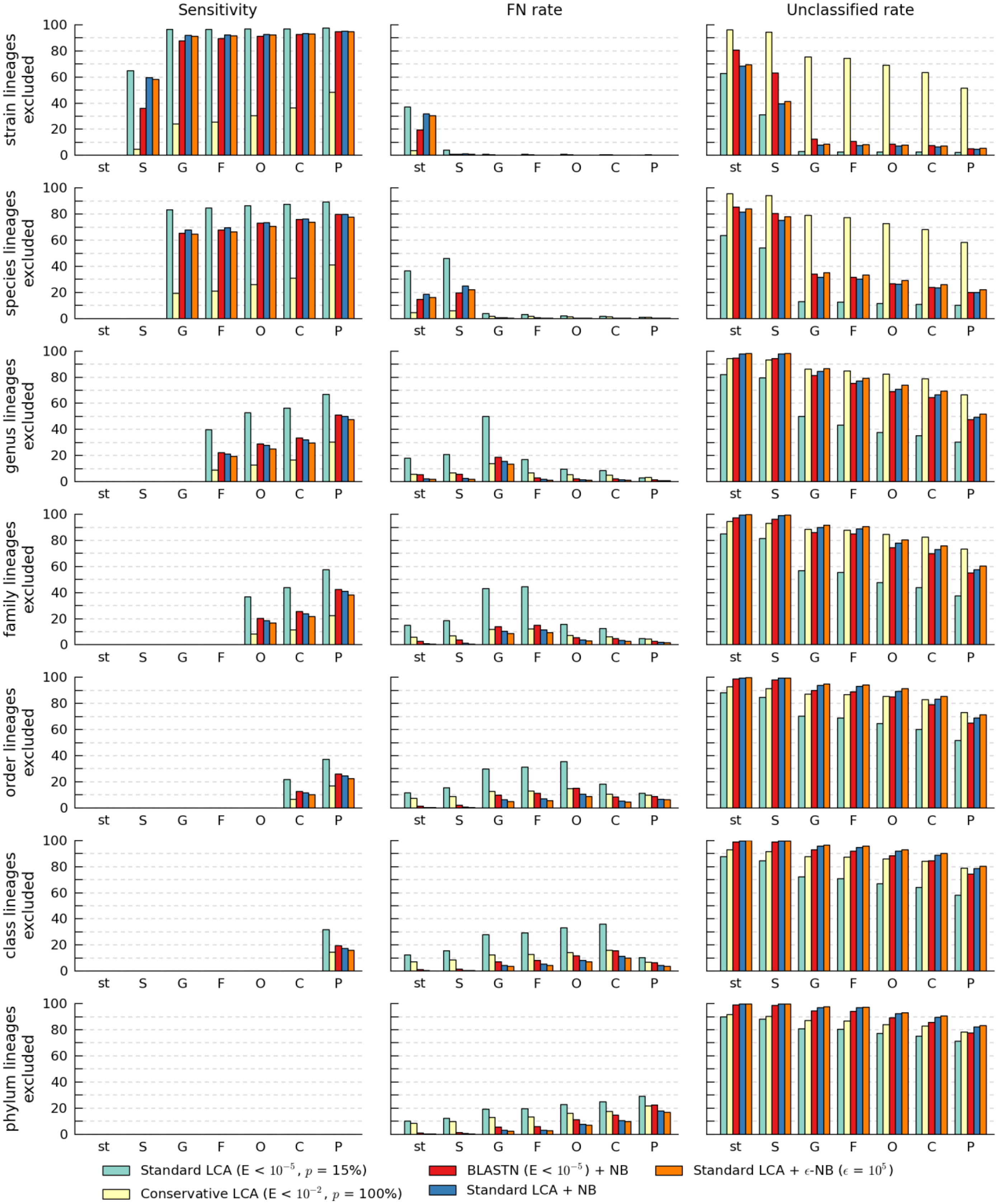

Figure S12. Absolute classification performance of rank-flexible classifiers over all 1000 bp query fragments using a leave-one-out evaluation framework. Fragments were considered correctly classified if they were assigned to the strain (st), species (S), genus (G), family (F), order (O), class (C), or phylum (P) of their source genome.
