# Supplementary material for: Classifying short genomic fragments from novel lineages using composition and homology
Source: BMC Bioinformatics. 2011 Aug 9;12:328. doi: 10.1186/1471-2105-12-328 (PMC3173459; doi:10.1186/1471-2105-12-328)
Supplement: Additional file 7 — Percentage of classified query fragments assigned to the correct rank, correct lineage, or incorrectly. Percentage of classified query fragments of length 400 bp (Additional file 7, Figure S13) and 1000 bp (Additional file 7, Figure S14) assigned to the correct rank, correct lineage, or incorrectly. [file 1471-2105-12-328-S7.PDF]

Additional File 7

Classifying short genomic fragments from novel lineages using composition and homology

Donovan H. Parks[1,§], Norman J. MacDonald[1,§], and Robert G. Beiko[1,*]

[1]Faculty of Computer Science, Dalhousie University, 6050 University Avenue, Halifax, Nova Scotia, Canada B3H 1W5

§ These authors contributed equally to this work.

[*] To whom correspondence should be addressed (beiko@cs.dal.ca).

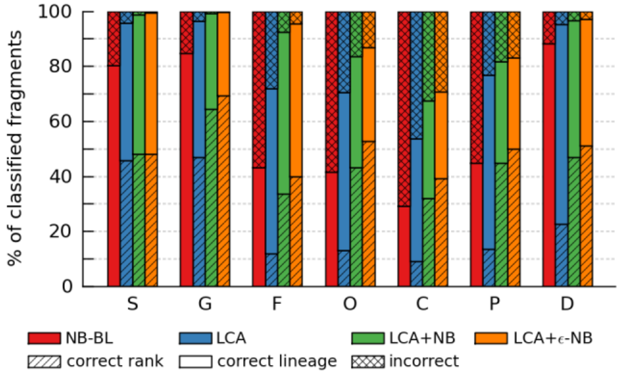

Figure S13. Percentage of classified query fragments of length 400 bp assigned to the correct rank, correct lineage, or incorrectly. Each set of bars indicates the performance at a given rank when the child lineages of that rank are excluded from the training set. For example, results at the genus level are calculated with species-level lineages excluded. Performance is reported at species (S), genus (G), family (F), order (O), class (C), phylum (P), and domain (D) ranks. The rank-specific NB-BL classifier always classifies query fragments at the strain level and as a result never assigns fragments to the correct rank. BLASTN and LCA results are for an E-value threshold of 10^{-5}. The LCA classifiers use *p*=15% and the ε-NB classifier uses $\varepsilon=10^5$.

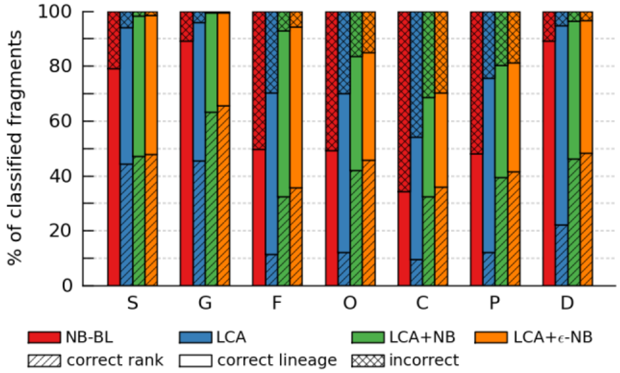

Figure S14. Percentage of classified query fragments of length 1000 bp assigned to the correct rank, correct lineage, or incorrectly. Each set of bars indicates the performance at a given rank when the child lineages of that rank are excluded from the training set. For example, results at the genus level are calculated with species-level lineages excluded. Performance is reported at species (S), genus (G), family (F), order (O), class (C), phylum (P), and domain (D) ranks. The rank-specific NB-BL classifier always classifies query fragments at the strain level and as a result never assigns fragments to the correct rank. BLASTN and LCA results are for an E-value threshold of 10^{-5}. The LCA classifiers use *p*=15% and the ε-NB classifier uses $\varepsilon=10^5$.
