# Supplementary material for: Classifying short genomic fragments from novel lineages using composition and homology
Source: BMC Bioinformatics. 2011 Aug 9;12:328. doi: 10.1186/1471-2105-12-328 (PMC3173459; doi:10.1186/1471-2105-12-328)
Supplement: Additional file 8 — Relative proportion of assigned reads from a glacier ice metagenome classified to different taxonomic groups. Profiles obtained using composition-based classifiers are contrasted with profiles obtained with classifiers making use of homology information. Taxonomic groups represented by less than 1% of the assigned reads are collectively represented as 'Other'. The proportion of assigned reads was 43% for BLASTN, 85% for NB, 21% for BLASTN+ε-NB with ε = 105, 46% for ε-NB with ε = 105, 13% for BLASTN+ε-NB with ε = 1010, and 22% for ε-NB with ε = 1010. BLASTN results are for an E-value threshold of 10-5. [file 1471-2105-12-328-S8.PDF]

Additional File 8

Classifying short genomic fragments from novel lineages using composition and homology

Donovan H. Parks[1,§], Norman J. MacDonald[1,§], and Robert G. Beiko[1,*]

[1]Faculty of Computer Science, Dalhousie University, 6050 University Avenue, Halifax, Nova Scotia, Canada B3H 1W5

§ These authors contributed equally to this work.
[*] To whom correspondence should be addressed (beiko@cs.dal.ca).

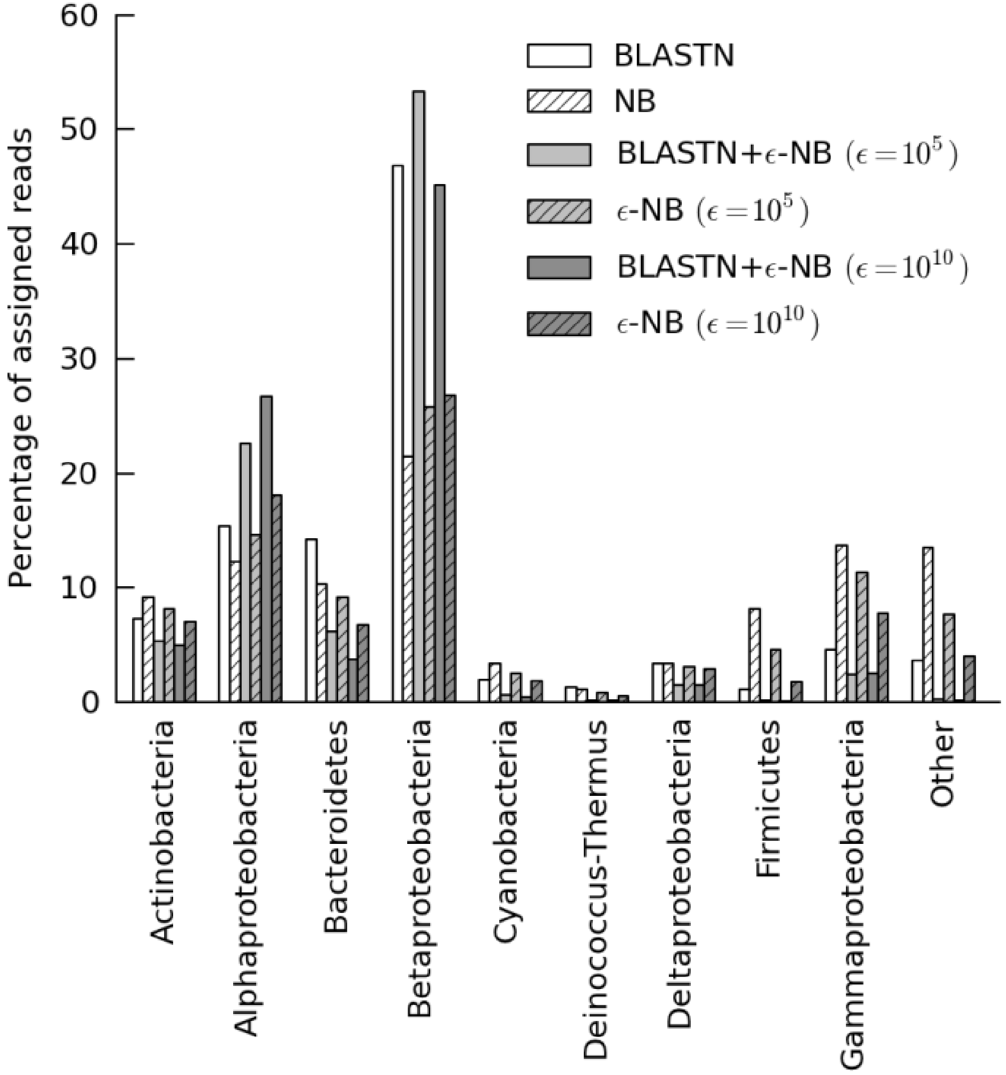

Figure S15. Relative proportion of assigned reads from a glacier ice metagenome classified to different taxonomic groups. Profiles obtained using composition-based classifiers are contrasted with profiles obtained with classifiers making use of homology information. Taxonomic groups represented by less than 1% of the assigned reads are collectively represented as ‘Other’. The proportion of assigned reads was 43% for BLASTN, 85% for NB, 21% for BLASTN+ε-NB with $\varepsilon=10^5$, 46% for ε-NB with $\varepsilon=10^5$, 13% for BLASTN+ε-NB with $\varepsilon=10^{10}$, and 22% for ε-NB with $\varepsilon=10^{10}$. BLASTN results are for an E-value threshold of 10^{-5}.
